# Deepening the Whole Transcriptomics of Bovine Liver Cells Exposed to AFB1: A Spotlight on Toll-like Receptor 2

**DOI:** 10.3390/toxins14070504

**Published:** 2022-07-20

**Authors:** Silvia Iori, Marianna Pauletto, Irene Bassan, Federico Bonsembiante, Maria Elena Gelain, Anisa Bardhi, Andrea Barbarossa, Anna Zaghini, Mauro Dacasto, Mery Giantin

**Affiliations:** 1 Department of Comparative Biomedicine and Food Science, University of Padua, Viale dell’Università 16, Legnaro, 35020 Padua, Italy; silvia.iori@phd.unipd.it (S.I.); marianna.pauletto@unipd.it (M.P.); irene.bassan@unipd.it (I.B.); federico.bonsembiante@unipd.it (F.B.); mariaelena.gelain@unipd.it (M.E.G.); mauro.dacasto@unipd.it (M.D.); 2 Department of Animal Medicine, Production and Health, University of Padua, Viale dell’Università 16, Legnaro, 35020 Padua, Italy; 3 Department of Veterinary Medical Sciences, Alma Mater Studiorum University of Bologna, Via Tolara di Sopra 50, Ozzano dell’Emilia, 40064 Bologna, Italy; anisa.bardhi@unibo.it (A.B.); andrea.barbarossa@unibo.it (A.B.); anna.zaghini@unibo.it (A.Z.)

**Keywords:** aflatoxin B1, bovine, liver, RNA-*seq*, oxidative stress, inflammatory response, toll-like receptor 2

## Abstract

Aflatoxin B1 (AFB1) is a food contaminant metabolized mostly in the liver and leading to hepatic damage. Livestock species are differently susceptible to AFB1, but the underlying mechanisms of toxicity have not yet been fully investigated, especially in ruminants. Thus, the aim of the present study was to better characterize the molecular mechanism by which AFB1 exerts hepatotoxicity in cattle. The bovine fetal hepatocyte cell line (BFH12) was exposed for 48 h to three different AFB1 concentrations (0.9 µM, 1.8 µM and 3.6 µM). Whole-transcriptomic changes were measured by RNA-*seq* analysis, showing significant differences in the expression of genes mainly involved in inflammatory response, oxidative stress, drug metabolism, apoptosis and cancer. As a confirmatory step, post-translational investigations on genes of interest were implemented. Cell death associated with necrosis rather than apoptosis events was noted. As far as the toxicity mechanism is concerned, a molecular pathway linking inflammatory response and oxidative stress was postulated. Toll-Like Receptor 2 (TLR2) activation, consequent to AFB1 exposure, triggers an intracellular signaling cascade involving a kinase (p38β MAPK), which in turn allows the nuclear translocation of the activator protein-1 (AP-1) and NF-κB, finally leading to the release of pro-inflammatory cytokines. Furthermore, a p38β MAPK negative role in cytoprotective genes regulation was postulated. Overall, our investigations improved the actual knowledge on the molecular effects of this worldwide relevant natural toxin in cattle.

## 1. Introduction

Aflatoxin B1 (AFB1) is a widespread mycotoxin produced by fungal species belonging to Aspergillus family (i.e., *A. flavus* and *A. parasiticus*). AFB1 occurrence is reported in several food and feed commodities, such as corn, wheat, peanuts, milk and eggs, posing a great potential risk to human and animal health [1]. As a matter of fact, aflatoxins (AFs) B1, B2, G1 and G2 represent a group of mycotoxins with hepatotoxic, genotoxic and carcinogenic effects [2]. Along this line, the International Agency for Research on Cancer (IARC) classified AFs as carcinogenic to humans (Group 1) [3]. Among them, AFB1 is considered as the most toxic AF, as well as one of the most potent liver carcinogen [4]. AFB1 contamination mainly affects agricultural crops from tropical and sub-tropical areas. However, greenhouse emission, global warming and precipitations also favour the spread of crop fungal infections in temperate regions. In this scenario, AFB1 crops contamination will likely rise in the next years, thereby becoming increasingly important in the European area [5,6].

The consumption of feed contaminated with AFB1 may affect health, as well as the performance of farm animals. In non-ruminant species such as poultry, the exposure to AFB1 negatively affects production traits like weight, egg production and hatchability [7,8,9]. In poultry and pigs, liver damage and immunosuppression were also reported, making them more vulnerable to diseases [10]. In cattle, AFs negatively affect production traits (e.g., milk, beef), growth and reproduction, as well as rumen metabolism [11,12].

AFB1 bioactivation and detoxification occur mostly in the liver by hepatic cytochrome P450s (CYPs), which belong to the phase I metabolizing enzymes, and by glutathione S-transferases (GSTs), a phase II family of conjugative enzymes, respectively [13]. In humans as well as other animal species, AFB1 is bioactivated by the CYP1A and CYP3A4 isozymes, giving rise to metabolic derivatives such as AFB1-8,9-exo-epoxide (AFBO). AFBO may form adducts with DNA, RNA and proteins, leading to toxicity, impairment of transcriptional and translational processes, together with carcinogenesis initiation [14].

AFB1 may also be converted in other toxic metabolites, such as the hydroxylated carcinogenic derivative Aflatoxin M1 (AFM1) and aflatoxicol (AFL), and relatively nontoxic metabolites like aflatoxin P1 (AFP1), aflatoxin Q1 (AFQ1) and aflatoxin B2a (AFB2a) [15]. The amount and nature of AFB1 metabolites depend on the CYP isozymes involved, which in turn differ among species, as well as to differences in concentration of hepatic GST isozymes [16]. In this respect, cattle are known to be more susceptible than horses or sheep [17,18]. Several aflatoxicosis outbreaks were reported in cattle, with consequent significant economic losses [19]. AFB1 toxicity depends also on other factors, such as age, duration and dose of exposure [20].

Given all the above-mentioned AFB1 adverse effects, it is undeniable that understanding the mechanistic toxicology of AFB1 in farm animals is a fundamental step in contributing to the management of the associated risk. Whole-transcriptome analysis is a powerful approach helping to reach this goal, providing a deeper look at the molecular pathways involved in hepatic AFB1 response [21]. As far as we know, only few RNA sequencing (RNA-*seq*) studies were conducted on livestock species. Specifically, in-vivo studies on the liver of poultry (i.e., turkey, ducklings) exposed to AFB1 and showing several hepatic disfunctions revealed the dysregulation of genes associated with drug metabolism, carcinogenesis, apoptosis, cell cycle, lipid metabolism and oxidation-reduction reactions [22,23].

Despite the cattle susceptibility to the toxic effects of AFB1, scarce information is available on the molecular mechanism governing AFB1 hepatotoxicity in cattle.

Only recently we demonstrated the occurrence of great transcriptional perturbations linked to inflammation, cellular damage and apoptosis pathways on a bovine fetal hepatocyte cell line (BFH12) exposed to 3.6 µM AFB1 [24]. Since this cell line is of fetal origin, thus characterized by a different drug metabolizing gene expression pattern compared to the adult one (e.g., weak CYP1A1 expression), we used 1 nM 3,3′,4,4′,5-pentachlorobiphenyl (PCB126) to increase CYP1A1 mRNA expression. Indeed, despite PCB126 pretreatment modulated the expression of a very low number of genes (*n* = 8), it affected both AFB1 metabolic pattern profile and the cytotoxicity rate [24].

In order to disclose AFB1 dose-dependent molecular effects without the influence of PCB126 pre-treatment, in the present study BFH12 cells were exposed for 48 h to increasing AFB1 sub-cytotoxic concentrations (i.e., 0.9 µM, 1.8 µM and 3.6 µM). Transcriptomic changes were measured by RNA-*seq* analysis. Then, confirmatory post-translational investigations on target proteins were executed. The expression of several genes related to inflammation and oxidative stress, together with cancer and drug metabolism, resulted dysregulated in a dose-dependent manner by the mycotoxin. Moreover, we postulated a putative pathway linking oxidative stress and inflammation in the response of cattle liver to AFB1 exposure.

Overall, the results obtained in the present study allowed a deeper characterization of AFB1 mechanistic toxicology in cattle liver, highlighting signaling pathways that could be promising targets in the treatment of aflatoxicosis.

## 2. Results

### 2.1. AFB1 Cytotoxicity

**Figure 1:**
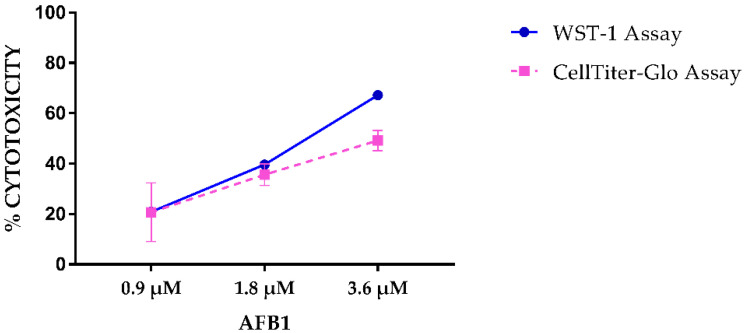
Cytotoxicity evaluation of increasing AFB1 concentrations in BFH12 cells using WST-1 and CellTiter-Glo^TM^ assays. Data are expressed as the mean percentage of dead cells relative to that of cells exposed to the vehicle only (0.1%) DMSO) ± mean standard error (SEM).

AFB1 cytotoxicity was estimated using two different metabolic assays, WST-1 and the CellTiter-Glo^TM^. Both tests showed a dose-dependent increase of AFB1 cytotoxicity (Figure 1). In particular, the percentage of dead cells obtained with WST-1 assay was 20.90%, 39.63% and 67.21% for AFB1 0.9 µM, 1.8 µM and 3.6 µM, respectively. Conversely, CellTiter-Glo^TM^ assay showed overall lower cytotoxicity values (20.72%, 35.66% and 49.18% for AFB1 0.9 µM, 1.8 µM and 3.6 µM, respectively). The cytotoxicity obtained with the highest concentration approximated the AFB1 IC_50_ estimated in our previous publication [24].

### 2.2. LC-MS/MS Quantification of AFB1, AFM1 and AFL

BFH12 cells metabolized AFB1, as they released AFM1 and AFL into the cell medium. Notably, the amount of such derivatives, as well as of AFB1, measured into the medium by means of LC-MS/MS, increased following a dose-dependent manner (Table 1).

### 2.3. Quantitation of Apoptosis and Necrosis by Annexin V and Propidium Iodide

**Figure 2:**
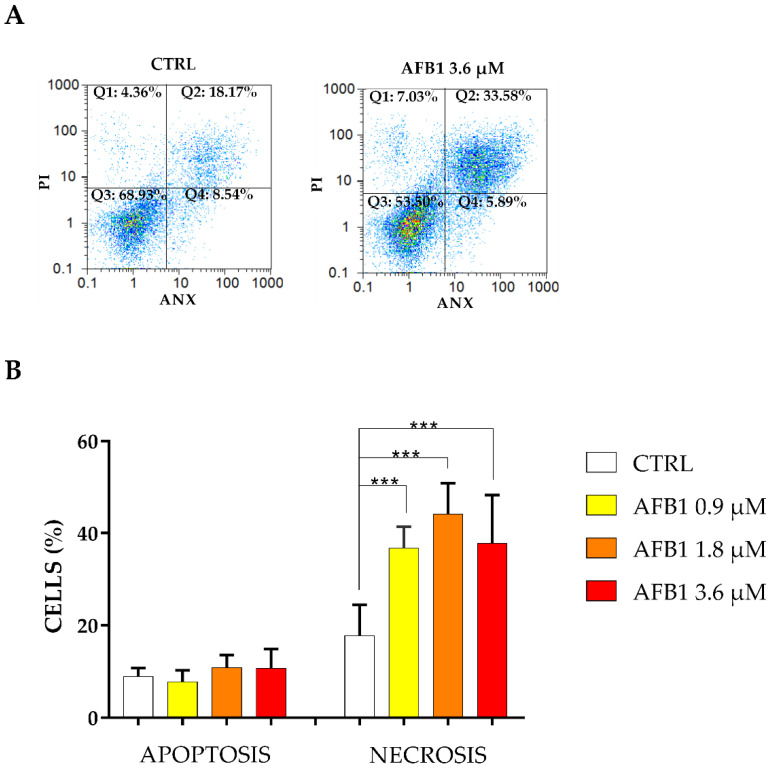
Assessment of apoptotic and necrotic cell rates by means of flow cytometry. (**A**) Scatter-plots of BFH12 cells exposed to 0.1% DMSO only (CTRL) or to 3.6 µM AFB1. ANX = Annexin V, PI = Propidium Iodide. Q1 and Q3 squares represent naked nuclei (negative to annexin V and positive to PI) and alive cells (negative to both annexin V and PI), respectively. Q2 square reports necrotic cells (positive to both annexin V and PI), and apoptotic cells are visible in Q4 square (positive to annexin V and negative to PI). The image is representative of six biological replicates. (**B**) Data are expressed as the mean ± SEM of six biological replicates (i.e., independent cell cultures), each one analyzed in triplicate. Statistical analysis: one-way ANOVA followed by Bonferroni’s multiple comparisons test; ***: *p* < 0.001, treated cells vs. control cells.

For the assessment of the cell death induced by AFB1, Annexin V/propidium iodide (PI) assay was used. The results obtained are shown in Figure 2. Overall, the cell death was primarily associated to necrosis; in this respect, statistically significant differences (*p* < 0.001) were obtained in all pair-wise comparisons between treated and control cells. Conversely, no statistically significant differences were obtained considering apoptotic rates.

### 2.4. Differential Gene Expression Analysis

**Figure 3:**
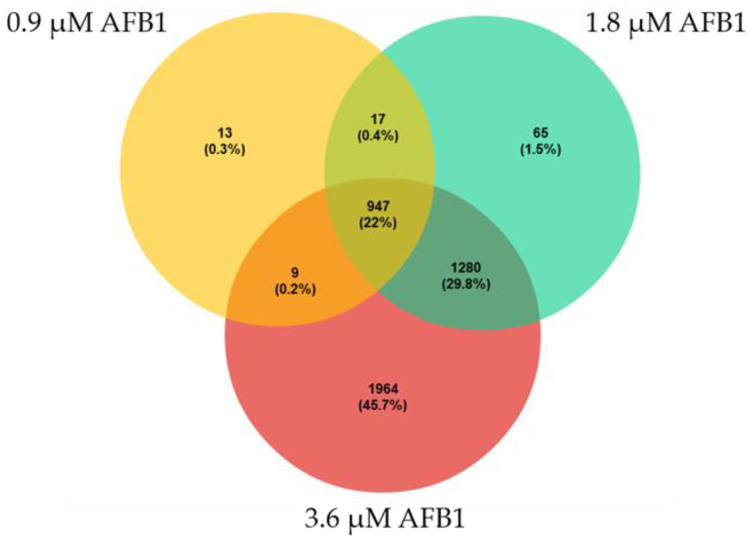
Venn diagram reporting number and percentages of unique and in common DEGs among the different AFB1 treatment conditions (i.e., 0.9 µM, 1.8 µM and 3.6 µM).

A total of 23,770,398 raw reads were obtained and deposited in GeneBank under the BioProject accession number ID PRJNA847423. All samples passed quality control measures for raw sequenced reads. After trimming, an average of about 24 million reads per sample were retained, with ~99% of reads mapping to the *B. taurus* reference genome. Numbers of raw reads passing the filters and reads mapping to the cow genome are provided in Table S1. The plot MDS (Figure S1) provided an unsupervised clustering of samples. The first dimension (*x* axis) clearly separated each experimental condition (i.e., CTRL, 0.9 µM AFB1, 1.8 µM AFB1 and 3.6 µM AFB1). Transcriptional changes in response to each AFB1 concentration were assessed through pair-wise comparisons between control and treated samples. In 0.9 µM AFB1-treated cells, a total of 986 DEGs was found, while 2309 and 4200 DEGs were highlighted in 1.8 µM AFB1- and 3.6 µM AFB1-treated cells, respectively (Figure S2). The whole list of DEGs resulting from each comparison is reported in Table S2. A Venn diagram was then constructed to visualize unique and shared DEGs after AFB1 treatments (Figure 3).

### 2.5. Functional and Gene Set Enrichment Analysis of DEGs

**Figure 4:**
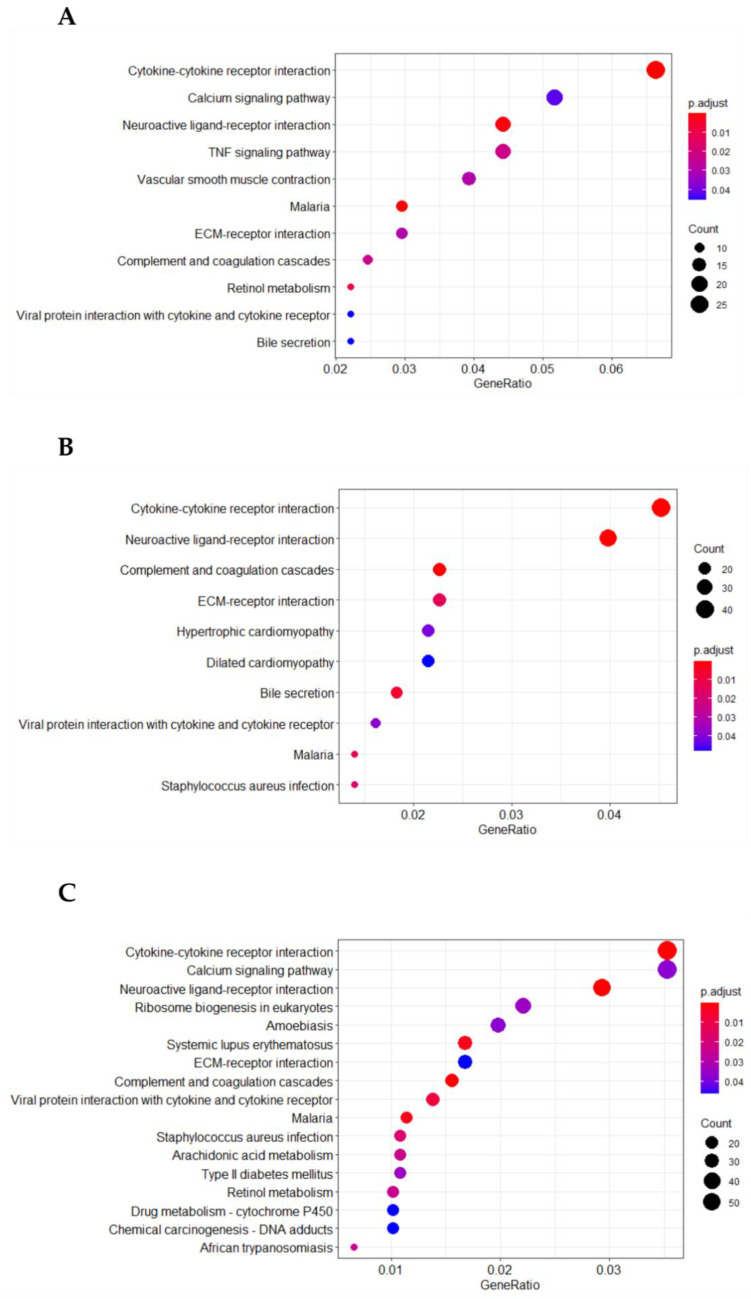
KEGG enrichment analysis of DEGs in 0.9 µM AFB1 (**A**), 1.8 µM AFB1 (**B**) and 3.6 µM AFB1 (**C**) treatments vs. control. Gene ratio is the percentage of DEGs over the total number of genes in a given pathway. Count (dot size) represents the number of DEGs enriched in a certain pathway.

The functional Kyoto Encyclopedia of Genes and Genomes (KEGG) pathway analysis was performed on DEGs modulated by AFB1 treatments. For each condition, the output of the analysis was reported in a dotplot with an adjusted *p*-value, highlighting 11, 10 and 17 enriched pathways for 0.9 µM, 1.8 µM and 3.6 µM AFB1 treatments, respectively (Figure 4, Table S3). Several pathways linked to inflammatory processes and immunity were found to be significantly over-represented (e.g., “Cytokine-cytokine receptor interaction”, “TNF signaling pathway” and “Complement and coagulation cascades”). Likewise, a number of pathways associated with pathogens infections (e.g., “Viral protein interaction with cytokine and cytokine receptor”, “Malaria”, “Staphylococcus aureus infection”, “Systemic lupus erythematosus”, “Ameobiasis” and “African trypanosomiasis”) were enriched; they were mainly represented by genes involved in inflammatory and immune response (e.g., interleukins, complement components, chemokines). Going deeper, several genes such as the prostaglandin-endoperoxide synthase 2 (PTGS2/COX2), the interleukin 6 (IL6), the CD40 and CD44 molecules, the toll-like receptor 2 (TLR2), the Fos proto-oncogene (FOS) and p38 MAPK family member β (MAPK11/p38β) increased their mRNA expression in a dose-dependent way as a consequence of the exposure. Conversely, the expression of few genes, e.g., the complement factor H (CFH), the C-X-C motif chemokine ligand 9 (CXCL9), 10 (CXCL10) and 11 (CXCL11), as well as the interleukin 33 (IL33) displayed a strong dose-related downregulation after AFB1 treatments.

The KEGG pathways related to drug metabolism (e.g., “Retinol metabolism” and “Drug metabolism-cytochrome P450”) were also over-represented. Worthy of note, the expression of several CYP family members resulted dysregulated by AFB1 treatment. More specifically, CYP1A1 showed a dose-dependent increase in its mRNA expression, while CYP2B6 gene expression resulted inhibited in a dose-dependent way. The aldehyde dehydrogenase 1 family members A1 (ALDH1A1) was also affected by AFB1, as its mRNA levels decreased as a consequence of the mycotoxin concentration; the same effect was observed for the UDP-glucuronosyltransferases family 1 member A1 (UGT1A1), the microsomal glutathione S-transferase 1 (MSGT1) and the flavin containing dimethylaniline monoxygenase 2 (FMO2).

KEGG enrichment analysis pointed out also the terms “Chemical carcinogenesis—DNA adducts”, “ECM-receptor interaction” and “Calcium signaling pathway”, which are all related to carcinogenesis processes. The list of genes whose expression resulted upregulated in parallel with the AFB1 concentration used includes, for example, lamin subunit gamma 2 (LAMC2), thrombospondin 1 (THBS1), cartilage oligomeric matrix protein (COMP), endothelin 1 (EDN1), secreted phosphoprotein 1 (PNS1) and neurotensin (NTS). Conversely, few genes such as collagen type IV alpha 5 (COL4A5) and 6 (COL4A6) chains, tenascin XB (TNXB), inositol 1,4,5-trisphosphate receptor type 2 (ITPR2) and calcium voltage-gated channel subunit alpha1 E (CACNA1E) showed an opposite behavior.

As far as KEGG GSEA analysis is considered, 23, 28 and 34 gene sets (GSs) were enriched in the cells incubated with 0.9 µM, 1.8 µM and 3.6 µM AFB1, respectively (Table S4). GSs shared among all AFB1 treatments were consistent with those highlighted with KEGG over-representation test. In fact, most pathways were related to inflammatory response and response to stress conditions (i.e., “NF-kappa B signalling pathway”, “P53 signalling pathway”, “Cell cycle”, “Salmonella infection”, “Toxoplasmosis” and “Epstein-Barr virus infection).

Additionally, the GS “Apoptosis” was reported. Looking deeply, the expression of genes such as the TNF Receptor Superfamily Member 10A (TNFRSF10A) and 10B (TNFRSF10A), together with the Growth Arrest and DNA Damage Inducible Alpha (GADD45A) and the Cell Death Inducing DFFA Like Effector B (CIDEB), showed an upregulation which was AFB1 dose-dependent. Moreover, the mRNA expression of the TNF Receptor Superfamily Member 10D (TNFRSF10D) and the Bcl-2-like protein 1 (BCL2L1) resulted in dysregulation, being inhibited by increasing concentrations of AFB1.

The log fold change (LogFC) of each gene mentioned above was reported in Table S5.

### 2.6. Protein-Protein Interaction (PPI) Network

**Figure 5:**
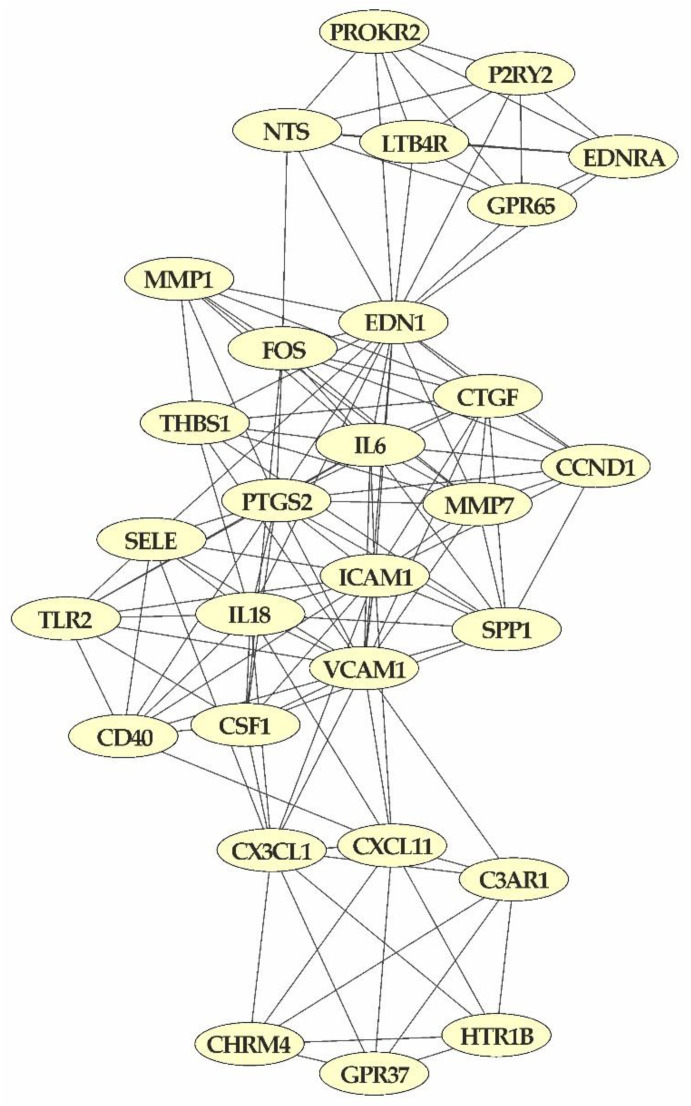
The most significant PPI module obtained with the analysis of the 947 DEGs in common among the three treatment conditions.

To investigate the molecular regulatory network of AFB1 at the protein level, the output of STRING database derived from the analysis of the 947 DEGs shared among all treatments (i.e., 0.9 µM AFB1, 1.8 µM AFB1 and 3.6 µM AFB1), was imported in Cytoscape software. The PPI network consisted of 738 nodes and 2193 edges. The top ten hub genes were implied in pathways related to inflammatory (IL6, CD44, PTGS2, FOS and the Secreted Phosphoprotein 1-SPP1) and carcinogenesis (Cyclin D1-CCND1, EDN1, Androgen Receptor-AR, G Protein Subunit Gamma 7-GNG7 and Connective tissue growth factor-CTGF) processes. According to the MCODE algorithm, 35 clusters were detected, and the module with the highest computed score (9.357) is shown in Figure 5. The KEGG pathway enrichment analysis of the module showed that the proteins were mainly associated with the inflammatory response (e.g., “TNF signaling pathway”, “Toll-like receptor signaling pathway”, “IL-17 signaling pathways” and “NF-kappa B signaling pathway”) as well as the carcinogenesis process (e.g., “PI3K-Akt signaling pathway”, “Pathways in cancer”, “Proteoglycans in cancer” and “Bladder cancer”) (Figure S4).

### 2.7. Inflammatory Response Signaling Pathway

**Figure 6:**
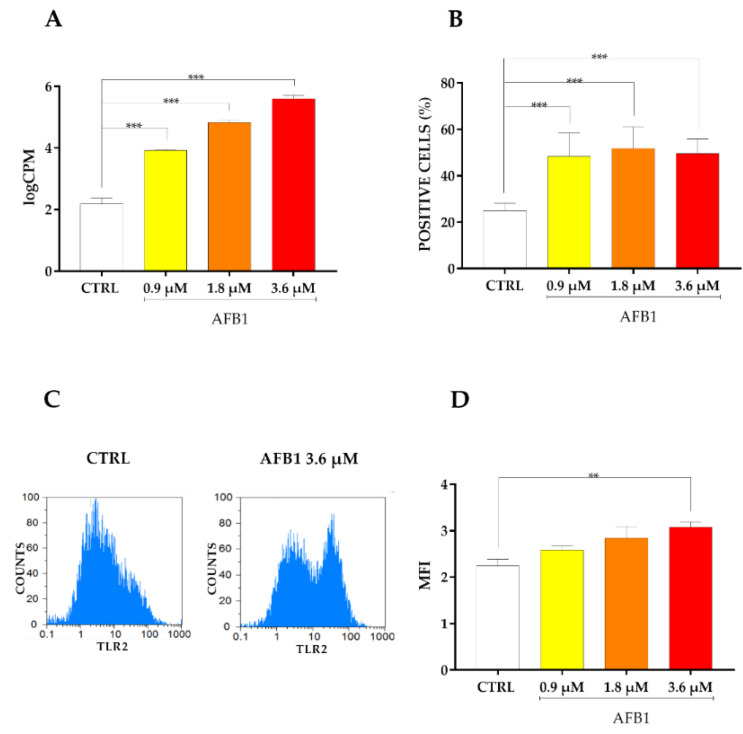
Effect of increasing doses of AFB1 (i.e., 0.9 µM, 1.8 µM and 3.6 µM) on TLR2 mRNA and protein expression. (**A**) Gene expression data are reported as the mean ± SEM of logarithm of counts per million (logCPM) relative to four biological replicates. (**B**) Cells positive (%) for TLR2 protein. Data are expressed as the mean ± SEM of six biological replicates, each one tested in triplicate. (**C**) Mean fluorescence intensity (MFI) of BFH12 cells exposed to 0.1% DMSO only (CTRL) and to 3.6 µM AFB1. The image is representative of six biological replicates. (**D**) Results of MFI analysis are expressed as the mean ± SEM of six biological replicates, each one tested in triplicate. Statistical analysis: one-way ANOVA followed by Bonferroni’s multiple comparisons test; **: *p* < 0.01 and ***: *p* < 0.001, treated cells vs. control cells.

Since RNA-*seq* analysis and PPI network displayed a strong enrichment of pathways related to inflammation, we decided to focus our attention on the inflammatory response pathways. In our experimental conditions, we observed a dose-dependent significant increase of TLR2 mRNA expression following AFB1 exposure (Figure 6A). Such an upregulation was confirmed also at the protein level using flow cytometry; in this respect, the percentage of TLR2 positive cells doubled in treated cells at all AFB1 concentrations (Figure 6B). Likewise, the MFI increased in a dose-dependent way in treated cells, showing a statistically significant boost at the highest dose (i.e., 3.6 µM, Figure 6C,D).

**Figure 7:**
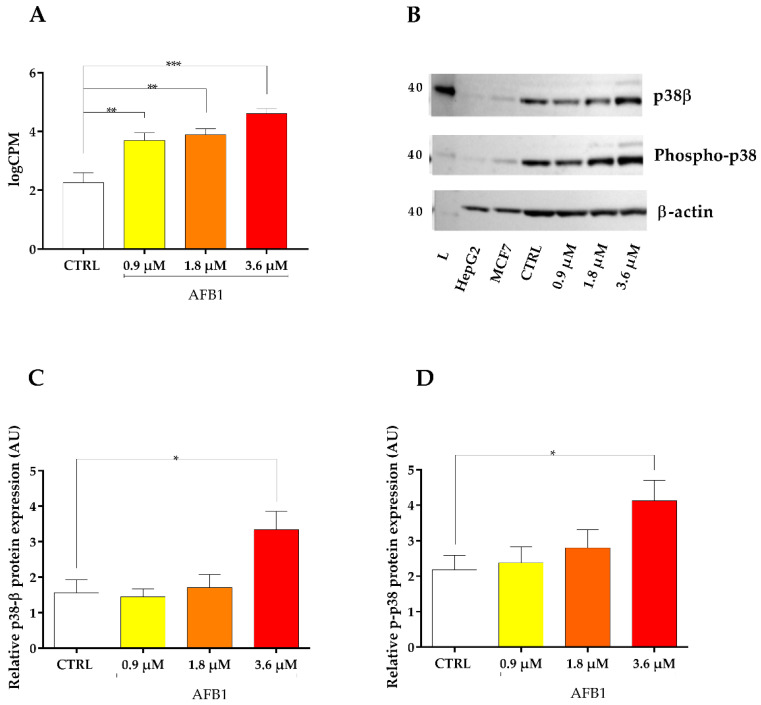
Effect of increasing doses of AFB1 (i.e., 0.9 µM, 1.8 µM and 3.6 µM) on p38β MAPK mRNA and protein expression (native and phosphorylated). (**A**) Gene expression data are reported as the mean ± SEM of logCPM relative to four biological replicates. (**B**) Immunoblotting of p38β and phospho-p38, MAPK using β-actin as loading control. The image is representative of six biological replicates. HepG2 and MCF7 cell lines were used as positive controls. (**C**,**D**) Densitometric analysis of p38β (**C**) and phospho-p38 (**D**) MAPK immunoblottings; data are expressed in arbitrary units (AU) as the mean ± SEM of six biological replicates. Statistical analysis: one-way ANOVA followed by Bonferroni’s multiple comparisons test; *: *p* < 0.05, **: *p* < 0.01 and ***: *p* < 0.001, treated cells vs. control cells.

In line with TLR2, p38β MAPK mRNA was significantly increased in a dose-dependent manner (Figure 7A). Immunoblotting investigations confirmed this trend, showing a gradual increase of p38β MAPK protein expression (both native and phosphorylated), which was statistically significant (*p* < 0.05) at the highest AFB1 concentration only (Figure 7B–D).

Moreover, enrichment analysis, as well as proteins interaction network, revealed the role of FOS in AFB1-mediated inflammatory induction (Figure S5A). Specifically, a dose-dependent mRNA induction (*p* < 0.001) was observed for FOS itself but also for other members of FOS family (i.e., FOSL1 and FOSB, Figure S5B,C), as well as for JUNB (Figure S6). Similarly, the Nuclear Factor Kappa B Subunit 2 (NFKB2) and the RELB Proto-Oncogene (RELB), two members of NF-κB family, exhibited a significant increase of mRNA expression (*p* < 0.001) in accordance with the AFB1 dose (Figure S7A,B).

**Figure 8:**
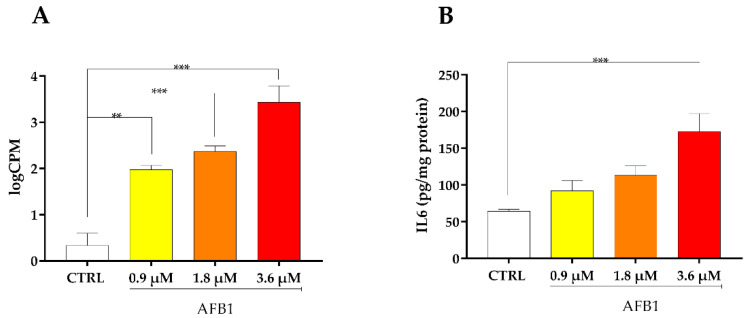
Effect of increasing doses of AFB1 (i.e., 0.9 µM, 1.8 µM and 3.6 µM) on IL6 mRNA and protein expression. (**A**) Gene expression data are reported as the mean ± SEM of logCPM relative to four biological replicates. (**B**) Amount of IL6 quantified by ELISA assay; data are expressed in picogram (pg) per milligram (mg) of total protein of six biological replicates, each one tested in duplicate. Statistical analysis: one-way ANOVA followed by Bonferroni’s multiple comparisons test; **: *p* < 0.01 and ***: *p* < 0.001, treated cells vs. control cells.

Among the top ten hub genes highlighted through the PPI network, IL6 displayed a significant increase of mRNA expression after the treatment with all AFB1 concentrations (Figure 8A). Likewise, AFB1-mediated induction of IL6 was confirmed at the protein level by means of ELISA assay, even if the increase in IL6 amount was significant (*p* < 0.01) at 3.6 µM AFB1 only (Figure 8B).

### 2.8. Oxidative Stress Signaling Cascade

**Figure 9:**
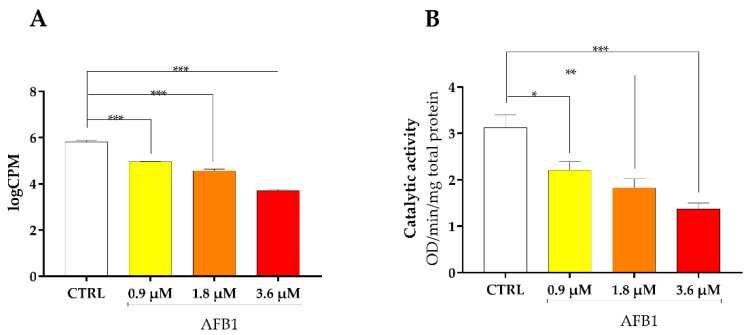
Effect of increasing doses of AFB1 (i.e., 0.9 µM, 1.8 µM and 3.6 µM) on NQO1 mRNA expression and catalytic activity. (**A**) Gene expression data are reported as the mean ± SEM of logCPM relative to four biological replicates. (**B**) Catalytic activity is expressed as median optical density (OD) per minute per mg of total protein ± SEM of four biological replicates, each one tested in duplicate. Statistical analysis: one-way ANOVA followed by Bonferroni’s multiple comparisons test; *: *p* < 0.05, **: *p* < 0.01 and ***: *p* < 0.001, treated cells vs. control cells.

Looking carefully at the outcome of the transcriptomic analysis, we found out the dysregulation of several genes related to oxidative stress response. Specifically, NQO1 (NAD(P)H Quinone Dehydrogenase 1) was significantly downregulated by AFB1 at all concentrations tested (*p* < 0.001, Figure 9A), and this inhibitory effect was dose-dependent. The same behavior was confirmed at the post-translational level, as AFB1 significantly inhibited NQO1 enzymatic activity starting from the lower concentration (*p* < 0.05, Figure 9B).

The detoxification enzyme UGT1A1 was affected by AFB1, too, but merely at the mRNA level (*p* < 0.001, Figure S8A–C), since immunoblotting data did not show any difference among the different experimental conditions.

**Figure 10:**
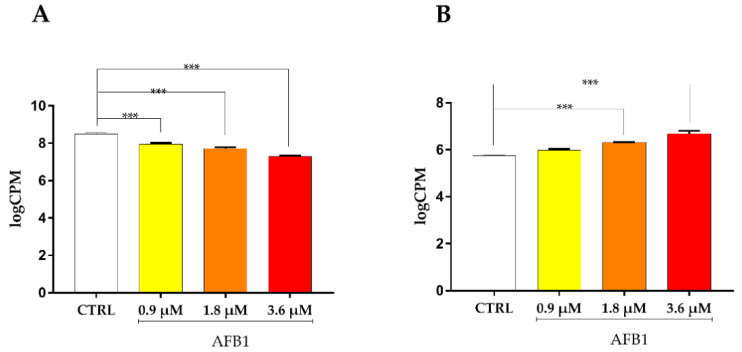
Effect of increasing doses of AFB1 (i.e., 0.9 µM, 1.8 µM and 3.6 µM) on NRF2 (**A**) and BACH1 (**B**) mRNA expression. Data are reported as the mean ± SEM of logCPM relative to four biological replicates. Statistical analysis: one-way ANOVA followed by Bonferroni’s multiple comparisons test; ***: *p* < 0.001, treated cells vs. control cells.

**Figure 11:**
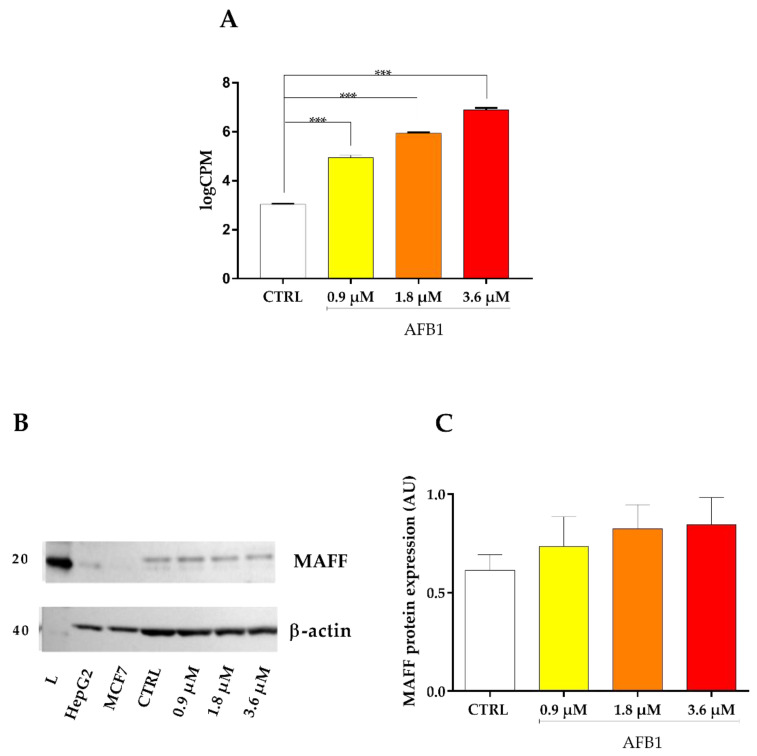
Effect of increasing doses of AFB1 (i.e., 0.9 µM, 1.8 µM and 3.6 µM) on MAFF mRNA and protein expression. (**A**) Gene expression data are reported as the mean ± SEM of logCPM relative to four biological replicates. (**B**) MAFF immunoblotting, using β-actin as loading control. The image is representative of six biological replicates. HepG2 and MCF7 cell lines were used as positive controls. (**C**) Densitometric analysis of MAFF immunoblottings; data are expressed in arbitrary units (AU) as the mean ± SEM of six biological replicates. Statistical analysis: one-way ANOVA followed by Bonferroni’s multiple comparisons test; ***: *p* < 0.001, treated cells vs. control cells.

With respect to the transcriptional regulators of cytoprotective genes, the master regulator NRF2 (Nuclear Factor Erythroid 2-Related Factor 2) was significantly deregulated by 1.8 and 3.6 µM AFB1 (*p* < 0.001, Figure 10A), while BACH1 (BTB and CNC homology 1), opposed to NRF2, being a repressor of genes involved in the oxidative stress response, was significantly up-regulated by 3.6 µM AFB1 only (*p* < 0.001, Figure 10B). Additionally, the MAFF (MAF BZIP Transcription Factors F), which is a basic region leucine zipper-type transcription factor involved in the binding of NRF2 and BACH1 to DNA [25], showed an increase of mRNA expression in accordance with the concentration used (*p* < 0.001, Figure 11A), while the protein expression evaluated by immunoblotting analysis highlighted a trend only (Figure 11B,C).

## 3. Discussion

So far, several studies investigated the molecular mechanism underneath AFB1 induction of hepatocellular damage in human and rat hepatocytes [25,26,27]. As to food-producing species, a fair amount of information about AFB1-mediated hepatotoxicity is available [22,23]; however, very little is known about AFB1 mechanistic toxicology in cattle liver. In the present study, the molecular mechanisms involved in the exploitation of AFB1 hepatotoxicity have been characterized in BFH12 cells, thereby improving the body of knowledge about the toxicological effects of this mycotoxin in this important farm animal species.

### 3.1. AFB1 Biotranformation

The metabolite profiling of AFB1, and particularly the production of the two main AFB1 derivatives AFM1 and AFL, was assessed in the cell medium after 48 h of incubation with the mycotoxin (0.9 µM, 1.8 µM and 3.6 µM). The amount of AFB1 and its derivatives increased proportionally to the AFB1 concentration used, thus proving BFH12 cells metabolize the mycotoxin. Present results, at least in part, confirm those previously obtained by [24]. Indeed, the amount of AFB1, AFM1 and AFL detected in BFH12 medium after 48 h of exposure to 3.6 µM AFB1, were higher than those here obtained. Nevertheless, in the former study cells had been pre-treated with a known CYP1A1 inducer (i.e., PCB126) to boost the cellular response to AFB1. Thus, it is conceivable to assume that the observed differences in the detectable concentrations of AFB1, AFM1 and AFL are attributable to the use of the PCB126, which most likely increased the capability of BFH12 to metabolize AFB1.

### 3.2. AFB1 Cytotoxicity and Mechanism of Cell Death

As already mentioned above, the AFB1 exposure and the resulting hepatotoxicity might substantially differ according to the species susceptibility, dose and time of exposure [20]. Consequently, selecting the optimum mycotoxin concentration to assess its in vitro toxic effects is quite challenging. The highest AFB1 concentration here used (3.6 µM) was chosen in accordance to our previous study [24], and it approximated the IC_50_ estimated in BFH12 cells after 48 h of incubation. Going further, a toxicokinetic study in which purified AFB1 was orally administered to cattle (0.35 mg/kg of body weight), reported a wide array of AF concentrations detected in tissues and plasma, with a maximum pick at 0.18 µM [28]. The lowest concentration tested in this study (i.e., 0.9 µM) is higher than the one mentioned above. Surely, it would be interesting to use realistic AFB1 concentrations; however, treating cells with an amount of AFB1 slightly higher may help to highlight the molecular mechanisms involved in the toxicity. Notably, the increasing AFB1 concentrations chosen in the present study were in accordance to those selected in previous cytotoxic experiments conducted in human and chicken hepatocytes, as well as in similar transcriptomic studies aimed at unveiling AFB1 mechanism of toxicity in chicken hepatocellular carcinoma (LMH) and HepG2 cell lines [29,30,31,32,33]. Overall, these data confirm AFB1 is cytotoxic to bovine hepatocytes, as previously reported [24].

Concerning the mechanism of cell death, apoptosis is a programmed death by which cells cease to grow and are phagocytized before undergoing membrane damage [34]. Conversely, necrosis is an uncontrolled mode of death by which cells reply to danger signals, like pro-inflammatory molecules, releasing cellular constituents after membrane rupture and leading to inflammation processes [35]. Previous studies on poultry species fed on diet contaminated with AFB1 (0.3–0.6 mg AFB1/basal diet) reported that AFB1 is likely to induce the hepatic programmed cell death [36,37,38]. In the present study, flow cytometry analyses showed that BFH12 cells exposed to 0.9 µM, 1.8 µM and 3.6 µM AFB1 exhibited a higher number of necrotic events compared to apoptotic ones. This experimental evidence was confirmed by transcriptomic results as several pathways linked to inflammation processes and apoptosis were enriched. Specifically, within apoptosis pathway, both pro-apoptotic and anti-apoptotic genes were induced by AFB1. Further details were reported below.

### 3.3. AFB1 Effects on BFH12 Cell Transcriptome

Overall, the whole-transcriptomic results suggest us that AFB1 deeply affects cattle hepatic transcriptome in a dose-dependent manner. This is clearly demonstrated by the number of DEGs in AFB1-exposed cells compared to control ones. Indeed, the number of DEGs increased in parallel with the AFB1 concentration. Notably, 947 DEGs (22%) were shared by the three different AFB1 concentrations; this leads us to hypothesize these genes represent a sort of core regulation mechanism of response to AFB1, which does not depend on the mycotoxin concentration. However, 13 (0.3%), 65 (1.5%) and 1964 (45.7%) genes were found to be specifically and differentially regulated by 0.9 µM, 1.8 µM and 3.6 µM AFB1, respectively. This would be indicative of an increasing cell responsiveness, with the involvement of specific genes, because of the use of increasing AFB1 concentrations. We decided to focus on DEGs shared among all the AFB1 conditions tested, with the goal of identifying a potential core molecular mechanism at the basis of AFB1 hepatotoxicity promotion.

In such a way, the Functional and Gene Set Enrichment Analysis of DEGs showed that most of the enriched pathways shared by all the AFB1 concentrations tested, and corresponding to the majority of genes modulated by the lowest concentration, encompasses genes associated with inflammation and immune responses. The output of PPI network analysis corroborated this result, as the list of the top ten hub genes included transcripts associated with inflammatory and immune response events. A further support of the main role of inflammation in AFB1-mediated hepatotoxicity was highlighted by the enrichment analysis of the protein interaction module, which resulted in several pathways linked to inflammatory processes. Previous studies conducted on poultry, mice and pigs confirmed the major role played by inflammation in AFB1 hepatotoxicity [39,40,41]. Thus, the effects of AFB1 on the inflammatory response are extensively discussed in a dedicated subsection.

Genes involved in carcinogenesis were also significantly affected by AFB1, as demonstrated by the KEGG enrichment and PPI network analysis. For instance, the pathway “ECM-receptor interaction” was enriched, which confirms what was previously reported in LMH cell line and turkey liver [32,42]. The extracellular matrix (ECM) consists of several macromolecules and mineral which are essential in maintaining cell architecture and functions; ECM proteins play a central role in this rearrangement by regulating complex cellular processes [43,44]. Therefore, the dysregulation of genes coding for ECM proteins may negatively affect cell network. Among them, laminins are crucial for cellular adhesion, differentiation and proliferation [45]. In our study, the LAMC2, a component of the laminin-332 protein, displayed an increased mRNA expression, which follows the AFB1 concentration used. This agrees with previous studies reporting an increased expression of LAMC2 in several cancer, such as hepatocellular and stomach carcinoma, as well as in lung cancer [46]. Another important ECM-structure glycoprotein whose mRNA level decreased after the treatment with the mycotoxin was the TNXB, an adhesion-modulatory ECM protein playing a crucial role in the cell structure organization [47]. Under pathological conditions, TNXB transcript dramatically decreased in most cancers, including hepatocellular carcinoma, thus corroborating our result [48]. Moreover, the gene expression of two collagenases, the COL4A5 and COL4A6, which are major components of the basement membrane [49] resulted dysregulated in the present study. Specifically, a decreased expression was observed, suggesting the disruption of the cell shape during cancer progression, as reported in previous studies [50,51]. AFB1 exposure also increased the expression of EDN1 in a dose-dependent way. EDN1 protein was also reported in the list of the top-ten hub genes deriving from the PPI network analysis, highlighting its important role in carcinogenesis process. The imbalance expression of EDN1 in cancer development is a well-known event. Indeed, EDN1 is a growth factor playing a critical role in tumorigenesis; it is frequently secreted in several tumors, such as liver, breast and colorectal cancers, in which enhances tumor growth by promoting angiogenesis [52,53,54,55,56].

The “Calcium signaling pathway” was another pathway which resulted significantly enriched in our study. The role of calcium in numerous cell processes, including cell proliferation, differentiation and even apoptosis, is well documented [57,58,59]. Consequently, the regulation of calcium channels may have a critical role in all the above listed cell functions. In our study, AFB1 reduced in a dose-dependent way the gene expression of the CACNAE1, a voltage-gated calcium channel (VGCC). It has been reported that a high expression of calcium channels is correlated with a reduction in cell proliferation capabilities [60]. Conversely, the downregulation of VGCC family genes, including the CACNAE1, is associated with the development and progression of several cancers [61].

It has been previously described that in human hepatoma cell lines AFB1 caused an imbalance between ROS production and antioxidant defense system, resulting into mitochondrial injury and finally into apoptosis [62,63]. In our study, KEGG GSEA analysis reported the over-representation of the “Apoptosis” term, which embraced both pro-apoptotic and anti-apoptotic genes. In poultry, the mycotoxin leaded to the overexpression of several hepatic death receptors, thus determining the induction of pro-apoptotic genes [36,37]. In our study, the death receptor genes TNFRSF10A and TNFRSF10B resulted in being upregulated in a dose-dependent way, following the exposure to AFB1. The upregulation of death receptor as a consequence of AFB1 exposure was previously reported also in pigs exposed to AFB1 in feed [42]. Moreover, our experiment highlighted an increase of expression of pro-apoptotic genes like GADD45A and CIDEB. The upregulation of such pro-apoptotic genes following the exposure to AFB1 was reported also in HepG2 and HepaRG cell lines, as well as in rat liver [64,65]. At the same time, our transcriptional results highlighted the upregulation of genes associated with anti-apoptotic functions like BCL2L1, a common anti-apoptotic protein that promotes cell survival, and TNFRSF10D, which plays an inhibitory role in TRAIL-induced cell apoptosis [66,67]. This last result would explain the outputs of flow cytometry investigations and the prevalence of necrotic events rather than apoptotic ones. Interestingly, the upregulation of BCL2L1 after AFB1 exposure was also observed in pig liver [42].

DEGs functional analysis found out, as expected, the enrichment of pathways associated with drug metabolism, too. As mentioned above, the CYP family plays a pivotal role in hepatic AFB1 biotransformation, and CYP1A and CYP3A subfamilies seemed to be those mostly involved. Moreover, it has been hypothesized that AFB1 might be a substrate for CYP2B6 in human [68,69]. In our study, CYP1A1 mRNA was significantly modulated by the treatment, as its expression increased in a dose-dependent way. Such a result is partially in contrast with previously published data. Indeed, in primary rabbit hepatocytes the mycotoxin inhibited CYP1A1 mRNA [70], while the inductive effect observed in the present study was reported also in primary human hepatocytes, in a rat hepatoma cell line, as well as in vivo in the liver of ducks fed with an AFB1-contaminated diet [71,72]. Our study also highlighted a strong dose-dependent decrease of CYP2B6 gene expression. By contrast, primary human hepatocytes exposed to AFB1 showed the overexpression of CYP2B6 gene [72]. Albeit contradictory, the observed gene dose-dependent downregulation could suggest that AFB1 is also a substrate of bovine CYP2B6, but such a hypothesis needs to be confirmed. Overall, in the present study a number of detoxifying enzymes was significantly inhibited by AFB1 in a dose-dependent manner. Among them there is ALDH1A1; besides its role in retinoic acid production, an involvement in the detoxification of lipid-derived toxic aldehydes, as well as in the metabolism of oxidative species, was previously described in rat liver [64,73]. Another important enzyme inhibited by AFB1 was MGST1, a membrane-bound enzyme displaying both conjugation and glutathione peroxidase functions, thus participating in the protection of cells against oxidative stress [74]. In mouse, rat and dog, MGST1 gene is expressed to a large extent in the liver; several endogenous and exogenous molecules may be substrate of this enzyme, including carcinogen drugs and products deriving from lipid peroxidation [75,76]. UGT1A1 gene encodes for an enzyme playing a major role in cellular protection. It is involved in the glucuronidation of both endogenous compounds and xenobiotics (e.g., dietary substances, hormones, environmental compounds and drugs) in more hydrophilic derivatives, thus facilitating the excretion with the urine and the bile [77]. Although UGT1A1 gene expression is not affected by AFB1 treatment in HepG2 and bovine mammary epithelial (BME) cell lines and in rat liver [78,79,80], in our study a strong decrease of UGT1A1 mRNA level was instead observed, suggestive of a peculiar response of cattle liver cells to AFB1 induction of oxidative stress. Interestingly, the expression of the abovementioned cytoprotective genes is regulated by NRF2 [81], a master regulator of the antioxidant response, whose mRNA expression was strongly decreased in the present study. Since oxidative stress has an outstanding role in hepatic initiation and progression of AFB1-induced toxicity, this pathway is discussed in depth below.

#### 3.3.1. AFB1-Mediated Induction of Inflammatory Response

To counteract the broad range of toxic and non-toxic insults occurring during life, mammalian species have evolved a network of complex defense mechanisms in which inflammatory response is a tightly controlled cellular process in response to harmful exogenous and endogenous stimuli [82]. TLRs are a family of pattern recognition receptors (PRRs) playing a pivotal role in innate immune response, as they enhance the inflammatory response by increasing cytokines production [83,84]. TLRs recognize a broad range of exogenous and endogenous ligands, including the pathogen-associated molecular patterns (PAMPs) and damage-associated molecular patterns (DAMPs) [83]. DAMPs are protein or non-protein molecules released by damaged and dying cells, even in the absence of pathogens [85]. Interestingly, danger signals deriving from oxidative stress and lipid peroxidation also seem to activate TLRs, triggering pro-inflammatory cytokines production [86,87]. Previous studies reported the involvement of the TLR family in the modulation of inflammatory and immune responses following AFB1 exposure in human and bovine species, as well as in chicken liver [88,89,90,91]. Moreover, in mice fed with a diet supplemented with AFB1, the mRNA levels of TLR2 in the liver increased, thus suggesting a possible role of TLR2 in mediating AFB1-stimulated inflammatory responses [41].

In the present study, a strong upregulation of TLR2 gene was noticed in BFH12 cells exposed to AFB1; hence, we speculate TLR2 could play a central role in AFB1-induced inflammatory response. Furthermore, this upregulation was also confirmed at the protein level by flow cytometry. Thus, it is conceivable to hypothesize that signalling proteins from dying cells, together with danger signals deriving from AFB1-induced oxidative stress, might activate the TLR2 cascade. In particular, the signal transduction through the activation of p38 MAPK family seems to be involved in the development of inflammation. Indeed, once activated, TLRs trigger an intracellular signaling cascade with the involvement of the p38 MAPKs, resulting in the nuclear translocation of certain transcription factors such as AP-1 and NF-κB and the consequent release of pro-inflammatory cytokines and chemokines [92,93]. Notably, an in-vivo experiment made in piglets confirmed the involvement of p38 MAPKs, AP-1 and NF-κB transcription factors in AFB1-induced liver injury [39]. NF-κB gene expression also resulted in being upregulated in the liver of chickens exposed to AFB1 through the feed [90]. In the present study, three members of the p38 MAPK family, as well as AP-1 and NF-κB transcription factors, were upregulated by AFB1. Then, we hypothesize AFB1 might induce the expression of pro-inflammatory cytokines in cattle liver through the involvement of the p38 MAPK pathway. In particular, the p38β gene was strongly induced in a concentration-dependent way. It is worth noting that such evidence was also confirmed at the protein level.

Additionally, in our study we observed a significant and concentration-dependent upregulation of IL6, both at the mRNA and protein level. In this respect, members of the interleukin family of pro-inflammatory cytokines have been proven to be involved in a wide array of cellular processes, including proliferation and differentiation [94]. Our results agreed with those previously published in broilers and pigs fed with a diet containing AFB1, and showing hepatic IL6 mRNA induction [39,40]. Interestingly, inflammatory cytokines are key players in CYP regulation, expression, and ultimately, catalytic activity [95]; specifically, IL6 has been shown to inhibit CYP2B6 [96,97,98]. As a matter of fact, in our experimental conditions we observed a strong and dose-dependent inhibition of the CYP2B6 gene (Figure S8A). Intriguingly, this result is in contrast with those observed in human hepatocytes exposed to AFB1, in which CYP2B6 gene was upregulated in a dose-dependent manner [72]. Therefore, it would be conceivable to hypothesize the presence of species-specific mechanisms of gene regulation activated by AFB1. Nevertheless, at protein level neither CYP2B6 expression nor catalytic activity corroborated RNA-*seq* results (Figure S8B–D), maybe suggesting the presence of post-translational (e.g., protein stabilization) mechanisms of gene regulation.

#### 3.3.2. AFB1-Mediated Induction of Oxidative Stress

In liver, AFB1-induction of hepatotoxicity is intimately linked to the ability of this mycotoxin in generating reactive oxygen species (ROS), thereby causing oxidative stress, which in turn triggers oxidative DNA damage and lipid peroxidation of membrane phospholipids [99,100]. ROS production is a common phenomenon in cells, which is normally counterbalanced by physiological defence mechanisms [11,12]. In this regard, NRF2 plays a critical role in the maintenance of cellular redox homeostasis by regulating a plethora of genes playing crucial role in cellular defence [101]. Hence, NRF2 activation by oxidative stress results in transcriptional induction of a battery of cytoprotective genes, such as proteins involved in the cellular antioxidant machinery (e.g., catalase—CAT, glutathione peroxidase—GPX and superoxide dismutase—SOD), as well as proteins involved in xenobiotic detoxification (e.g., NQO1, UGT, GST and ALDH) [12,13,14,15,81,102]. Specifically, under unstressed condition, NRF2 is associated to Kelch-like ECH-associated protein 1 (KEAP1), which leads to NRF2 ubiquitination and consequently to its proteasomal degradation. In presence of oxidative stress stimuli, the negative regulator KEAP1 is arrested, and NRF2 translocates into the nucleus where it forms a heterodimer with the sMAF [103].

This heterodimer can bind to the Maf (musculoaponeurotic fibrosarcoma) protein recognition element (MARE), leading to transactivation of cytoprotective genes [84]. Another important transcription factor involved in oxidative stress response is BACH1, which competes with NRF2 for binding to MARE. Notably, once BACH1-sMAF heterodimer is formed, the interaction with MARE inhibits the transcription of many oxidative stress-response genes, thus acting as a transcriptional repressor [104]. Besides heterodimers with NRF2 and BACH1, sMAF can form homodimers, acting as transcriptional repressor of antioxidant genes transcription [105].

In-vivo studies conducted on rat liver reported that AFB1 can impair the fine-tuning defence mechanism by inhibiting the NRF2 protein, enhancing the production of free radicals and lipid peroxides, thereby causing hepatocellular damage [25,106]. In line with these findings, the downregulation of NRF2 and downstream cytoprotective genes was shown also in broilers fed with AFB1-contaminated diet [107]. Moreover, an in-vivo study conducted on mice orally administrated with AFB1 showed a significant decrease of NRF2 as well as of antioxidant-related genes expression in the liver [108]. The same NRF2 downregulation pattern was shown, both at the mRNA and protein level, in the ileum of ducks fed on an AFB1-contaminated diet [109]. Likewise, we previously reported [24] and we confirmed in the present study the inhibition of NRF2 mRNA expression in bovine hepatocytes incubated with AFB1. Consistently with these findings, in the present study NRF2 mRNA was inhibited by AFB1. Interestingly, in the present study, KEAP1 did not show any significant modulation; this allows us to hypothesize an alternative mechanism of NRF2 regulation, possibly KEAP1-independent (e.g., protein kinase phosphorylation cascade, interaction with other proteins), which needs to be further investigated.

Unlike NRF2, in the present study, BACH1 gene expression was upregulated. As with sMAF, we noticed that MAFF was induced by AFB1 both at the mRNA and protein level. Overall, these findings suggest a transcriptional inhibition of cytoprotective genes as a result of AFB1 exposure. In this respect, looking at the RNA-*seq* output, we noticed the downregulation of several cytoprotective genes whose expression is regulated by NRF2. Among them, there is NQO1, a FAD-dependent flavoprotein involved in two-electron reduction of quinone to hydroquinone, thus avoiding the formation of semiquinones and ROS [110]. We measured the antioxidant capacity of this enzyme, and observed a concentration-dependent inhibition of the catalytic activity, confirming the NQO1 inhibition noticed at the protein level. Another important cytoprotective gene whose expression resulted downregulated in a dose-dependent way was the ALDH1A1. Furthermore, p38 MAPKs, in addition to the role in the inflammatory response, seem to also be involved in the antioxidant and cytoprotective responses. In particular, they act as repressors of genes directly involved in the oxidative stress response, whose expression is regulated by NRF2 [111,112]. As an example, it has been reported that UGT1A1 promoter contains one functional ARE that is responsible for the NRF2-dependent induction [112]. In our study, UGT1A1 mRNA expression appeared to be downregulated by AFB1 exposure in a concentration-dependent manner. Similar to CYP2B6, no significant differences in UGT1A1 protein amount were obtained in AFB1-exposed cells; this evidence could be suggestive of a protein stabilization mechanism counteracting UGT1A1 mRNA level decrease. It is also conceivable to suppose a contribution of p38 MAPK pathway in the downregulation of cytoprotective genes, leading to an increase of oxidative stress events.

**Figure 12:**
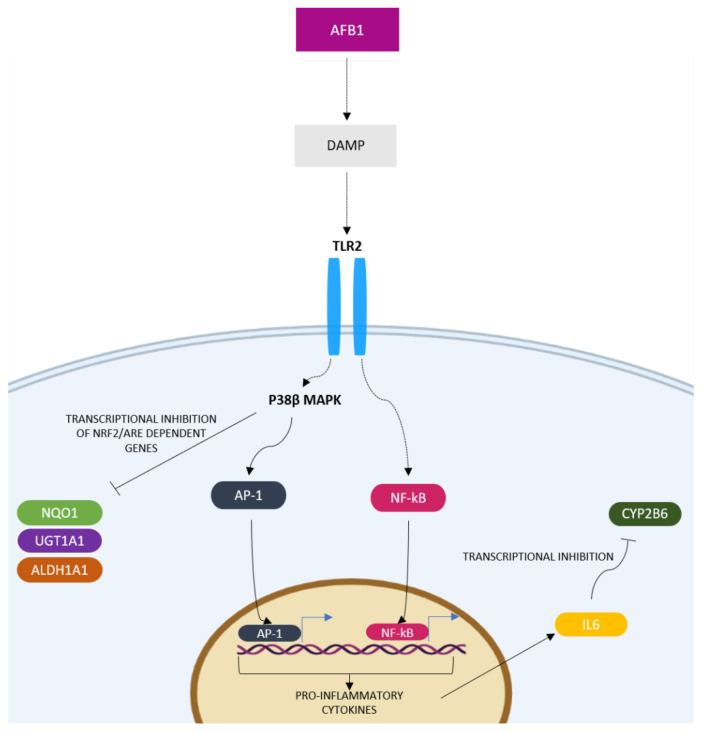
AFB1 induction of hepatotoxicity in BFH12 cells. Danger signals deriving from oxidative stress, as well as the DAMP, lead to TLR2 activation which triggers a downstream signaling cascade with the involvement of p38β MAPK. In turn, p38β MAPK allows the nuclear translocation of AP-1 and NF-kB transcription factors, thus leading the release of IL6 pro-inflammatory cytokine. In addition, p38β MAPK induction negatively regulates the expression of NRF2-dependent cytoprotective genes (e.g., NQO1, UGT1A1 and ALDH1A1), while IL6 inhibited CYP2B6 mRNA expression.

Figure 12 graphically described the putative connection between inflammation and oxidative stress pathways induced by AFB1 exposure in BFH12 cells, highlighting the central role of TLR2 in hepatotoxicity induction.

## 4. Conclusions

Following the need to increase our knowledge on molecular mechanisms underlying AFB1 hepatotoxicity in cattle, the fetal bovine hepatocytes cell line (BFH12) was exposed to increasing AFB1 concentrations (i.e., 0.9 µM, 1.8 µM and 3.6 µM), and then whole-transcriptomic analysis and post-translational confirmatory assays were implemented. AFB1, biotransformed by BFH12 cells, induced cell death via necrosis rather than apoptosis, and deeply affected cattle transcriptome in a dose-dependent manner, as the number of DEGs increased according to AFB1 concentration used. Specifically, the 22% of DEGs was shared among all the three AFB1 concentrations, suggesting a core molecular mechanism, independent from the dose used. Several pathways were affected by AFB1 (e.g., cancer, apoptosis and drug metabolism), including inflammation and oxidative stress. Based on transcriptomic data and post-translation investigations, a putative molecular signaling pathway linking AFB1 to oxidative stress and inflammation response events was proposed. Specifically, TLR2 activation seems to play a central role in AFB1-mediated exploitation of hepatotoxicity by triggering an intracellular signaling cascade, which in turn promotes a pro-inflammatory response. Overall, the present study contributes to gain new insights into the mechanistic toxicology of AFB1 in bovine liver.

## 5. Materials and Methods

### 5.1. Chemicals and Reagents

AFB1 (from *A. flavus*; CAS Number 1162-65-8), dimethyl sulfoxide (DMSO), dexamethasone, insulin from bovine pancreas and trypan blue solution were obtained from Sigma-Aldrich (St. Louis, MO, USA). AFB1 (purity 99.9%) and AFM1 (purity 99.1%) was purchased from Lab Service Analytica (Bologna, Italy). AFL (purity 98%) and 13C17-AFB1 (purity 98%) were obtained from DBA Italia (Milano, Italy) and Orsell (Modena, Italy), respectively. The WST-1 Cell Proliferation Reagent was from Roche (Basel, Switzerland), whereas P450-Glo™ CYP2B6 and CellTiter-Glo^®^ Luminescent Cell Viability Assay kits were from Promega Corporation (Madison, WI, USA). The RNeasy Mini kit was from Qiagen (Hilden, Germany). The Bovine IL6 Elisa kit (MBS733925) was purchased from MyBioSource (San Diego, CA, USA). NQO1 Activity Assay kit (ab184867) and rabbit anti-CYP2B6 (ab69652) were from Abcam (Cambridge, UK). The rabbit anti-rat/mouse/human UGT1A1 (AB10339) was from Sigma-Aldrich (St. Louis, MO, USA). The mouse anti-human p38β MAPK11 (F-3; sc-390984) and rabbit anti-human p-p38 MAPK (E-1; sc-166182) were obtained from Santa Cruz Biotechnology (Dallas, TX, USA). The rabbit anti-human MAFF (PA5-85462), Annexin V-FITC Apoptosis Detection Kit (BMS500FI), the Accutase Enzyme Cell Detachment Medium (00-4555-56) and the BCA assay kit were purchased from Invitrogen, Life Technologies (Carlsbad, CA, USA). The rabbit anti-beta actin (ACTB, GTX109639) and the goat anti-mouse IgG (GTX213111-01) were from GeneTex (Irvine, CA, USA). The human anti-bovine CD282:FITC (AbD12542) and HuCAL Fab-dHLX-MH Negative Control antibody (AbD04652) were purchased from Bio-Rad (Hercules, CA, USA). Analytical standards of AFB1, AFL, AFM1 and AFB2 were purchased from DBA Italia (Milano, Italy). All solvents used for analysis were of LC–MS grade: acetonitrile and formic acid were from Sigma Aldrich (Milano, Italy), ultrapure water was freshly produced in-house (Millipore, Milano, Italy).

### 5.2. Cell Culture

The bovine SV40 large T-antigen-transduced fetal hepatocyte-derived cell line (BFH12) [113] was provided by Dr. Axel Schoeniger (Institute of Biochemistry, University of Leipzig, Leipzig, Germany). Conditions of cell line maintenance have already been reported in [24].

### 5.3. AFB1 Cytotoxicity

Cells were seeded in 96-well flat-bottom plates at a density of 6 × 10^3^ cells/well, and were exposed for 48 h to three AFB1 concentrations (0.9 µM, 1.8 µM and 3.6 µM) dissolved in 0.1% DMSO as a vehicle. Cells exposed to 0.1% DMSO were used as control. AFB1 concentrations were selected based on the dose-response curve defined in our previous study [24]; specifically, the chosen AFB1 concentrations were sub-cytotoxic and below the IC_50_ (the mycotoxin concentration inhibiting cell viability by 50%). At the end of the incubation time, BFH12 viability was determined using the WST-1 Cell Proliferation Reagent and CellTiter-Glo^TM^ cell viability assay kit, following manufacturers’ instructions. Four and six independent cell cultures (i.e., biological replicates) per experimental group were performed, respectively. Each concentration was tested in sextuplicate.

### 5.4. Cells Incubation for Gene Expression Analysis, Post-Translational and Analytical Investigations

To evaluate the effects of increasing AFB1 concentrations on the whole BFH12 transcriptome, cells were seeded on 6-well culture plates at a density of 5 × 10^4^ cells/well and exposed to the abovementioned AFB1 concentrations, or the vehicle only, for 48 h, as reported in Section 5.3. At the end of the incubation time, the medium was collected and stored at −80 °C for analytical investigations, while cells were washed with 1X PBS/EDTA, re-suspended in 600 µL of RLT buffer (Qiagen) containing 6 µL of β-mercaptoethanol, vortexed and stored at −80 °C until use. Four independent experiments were executed.

For the evaluation of apoptosis and necrosis phenomena, as well as to isolate proteins for downstream analyses (i.e., immunoblotting, ELISA, flow cytometry), cells were seeded in Petri dishes (90 mm diameter) at a density of 3 × 10^5^ cells/Petri dish, and exposed either to AFB1 or DMSO following the same experimental protocol described above. Six independent experiments were executed.

### 5.5. LC-MS/MS Quantification of AFB1, AFM1 and AFL

The amount of AFB1 and its metabolites (AFM1 and AFL) was measured in the medium of all experimental conditions by LC-MS/MS. Samples were thawed at room temperature, mixed by vortexing for 30 s, and centrifuged at 15,000× *g* for 10 min. Twelve µL of the supernatant was mixed with 1.5 mL of a 0.1% formic acid in water:acetonitrile 85:15 (*v*/*v*) solution, also containing the internal standard AFB2, and then 5 µL was injected in the system. The LC was a Waters Acquity UPLC binary pump, equipped with an Acquity BEH C18 (50 × 2.1 mm, 1.7 µm) reversed-phase column, kept at 40 °C (Waters, Milford, MA, USA). Chromatographic separation was obtained in a 4 min run under programmed conditions, with a variable mixture of 0.1% formic acid in water and acetonitrile flowing at 0.3 mL/min. The detector was a Waters Xevo TQ-S Micro triple quadrupole mass spectrometer (Waters, Milford, MA, USA), operating in positive electrospray ionization (ESI+) with 3.0 kV capillary voltage. Source temperature was 150 °C and desolvation temperature was 600 °C; desolvation and cone gas flow were 900 and 50 L/h, respectively. For each analyte, the following specific transitions (with relative Cone Voltage and Collision Energy values) were monitored: 313.17 > 241.12 *m*/*z* (CV 80 V; CE 34 eV) and 313.17 > 284.90 *m*/*z* (CV 80 V; CE 20 eV) for AFB1; 329.17 > 273.08 *m*/*z* (CV 70 V; CE 24 eV) and 329.17 > 229.11 *m*/*z* (CV 70 V; CE 38 eV) for AFM1; 297.15 > 141.04 *m*/*z* (CV 78 V; CE 48 eV) and 297.15 > 115.01 *m*/*z* (CV 78 V; CE 50 eV) for AFL; 315.13 > 259.03 *m*/*z* (CV 70 V; CE 26 eV) and 315.13 > 287.06 *m*/*z* (CV 70 V; CE 24 eV) for AFB2. Data acquisition and analysis was carried out with MassLynx 4.2 software (Waters, Milford, MA, USA).

### 5.6. Total RNA Extraction and RNA-seq Analysis

Total RNA was isolated using RNeasy Mini kit following the manufacturer’s instructions. Subsequently, the quali-quantitative evaluation of total RNA was performed as described elsewhere [24]. For each experimental condition (i.e., CTRL, 0.9 µM AFB1, 1.8 µM AFB1 and 3.6 µM AFB1), four independent biological replicates were considered. Library construction and sequencing with NextSeq 500 system were performed at Centro di Ricerca Interdipartimentale per le Biotecnologie Innovative (CRIBI; University of Padova). Reads processing (i.e., quality check, trimming, filtering out and mapping against *Bos taururs* reference genome) was performed, as previously described [24]. Differential expression analysis was handled in EdgeR [114] on samples grouped in accordance with the treatment condition. Pair-wise comparisons between each treatment condition and the control were carried out to highlight transcriptional changes induced by increasing concentration of AFB1 (i.e., 0.9 µM AFB1 vs. CTRL, 1.8 µM AFB1 vs. CTRL, 3.6 µM AFB1 vs. CTRL), setting FDR at <0.05. ClusterProfiler package [115] was then implemented in R environment to functionally interpret significant differentially expressed genes (DEGs) through KEGG over-representation test and KEGG Gene Set Enrichment Analysis (GSEA) [116], as described in [117]. Expression of genes of interest was reported as logarithm of counts per million (logCPM), with a *p*-value threshold set at ≤0.05.

### 5.7. PPI Network Analysis

PPI network of the 947 DEGs shared among all treatments (i.e., 0.9 µM AFB1, 1.8 µM AFB1, 3.6 µM AFB1) was constructed using the online database Search Tool for the Retrieval of Interacting Genes/Proteins (STRING) with default parameters (https://string-db.org/cgi/input.pl, accessed on 5 June 2021). To better visualize the PPI network, the output data were then imported in Cytoscape software (version 3.8.2; https://cytoscape.org/, accessed on 5 June 2021) setting Degree Filter > 10. The Cytoscape plug-in Molecular Complex Detection (MCODE) was implemented to detect modules with the following parameters: Degree cutoff = 10, Node Score Cutoff = 0.2 and *K*-Core = 2. KEGG over-representation test was then implemented on genes belonging to the module with the highest score.

### 5.8. Flow Cytometry

The rate of necrosis and apoptosis induced by AFB1, as well as the protein expression of TLR2, were determined by flow cytometry. Six independent experiments (six independent cell cultures) were executed and each biological replicate (i.e., CTRL, 0.9 µM AFB1, 1.8 µM AFB1 and 3.6 µM AFB1) was analyzed in triplicate. After 48 h of incubation with the mycotoxin, cells were detached using Accutase and centrifuged at 1100 rpm for 10 min at 4 °C. Then, for the assessment of necrosis and apoptosis, the Annexin V-FITC Apoptosis Detection Kit was used following the manufacturer’s instruction. Necrotic cells were positive for both Annexin-V FITC and PI, while apoptotic cells were positive for Annexin-V FITC only.

For TLR2 protein quantification, 500,000 cells were harvested and resuspended in 500 μL of RPMI 1640 medium containing sodium azide and FBS, to reach a concentration of 1000 cells/μL. Cells (50,000/tube) were incubated for 1 h at 4 °C with the human anti-bovine TLR2 monoclonal antibody (dilution 1:100) and resuspended in 900 µL of PBS for data acquisition. In both cases, CyFlow Space flow cytometer (Partec-System, Sysmex Europe GmbH, Norderstedt-Amburgo, Germany) was used and data were analyzed with FloMax software (version 2.82). For each replicate, 20,000 events were acquired. The morphology and the complexity of cells were evaluated in forward scatter (FSC) and side scatter (SSC), while TLR2-positive cells were identified on fluorescence channel 5 versus FSC.

### 5.9. Protein Isolation and Immunoblotting

After collection of the culture medium, monolayers were washed with the lysis buffer (TrisHCl 20 mM, pH 7.4) and scraped off with 500 µL of the same buffer. The cell suspension was then subjected to repeated freeze–thaw cycles in liquid nitrogen and in a water bath (37 °C), respectively. The cell debris was removed by centrifugation at 12,000× *g* for 10 min (4 °C), and the supernatant was collected. Protein content was quantified using the BCA assay kit following the manufacturer’s instructions. Fifteen µg of total proteins were separated on NuPAGE^®^ Novex^®^ 4–12% Bis–Tris Gels by using the XCell SureLock™ Mini-Cell electrophoresis system (Invitrogen, Eugene, OR, USA), and then transferred onto nitrocellulose filters as previously described [118]. Two human positive controls (proteins isolated from HepG2 and MCF7 human cell lines) were loaded on each minigel. Membranes were incubated with anti-ACTB (1:6000 final concentration, 2 h), p38β MAPK11 and p-p38 MAPK (1:1000, overnight), CYP2B6 (1:500, overnight), MAFF (1:1000, overnight) and UGT1A1 (1:2000, overnight) primary antibodies, then with horseradish peroxidase-conjugated goat anti-rabbit (1:6000, 1.5 h, for ACTB, CYP2B6, MAFF, UGT1A1) or anti-mouse XX (1:1000, 1.5 h, for p38β MAPK11 and p-p38 MAPK) IgG. The specific proteins were detected by using SuperSignal^®^ West Pico chemiluminescence substrate (Pierce, Life Technologies, Foster City, CA, USA) according to the manufacturer’s instructions. Immunopositive bands were captured by the iBright Imaging Systems (iBright FL1500, Thermo Fisher Scientific, Waltham, MA, USA) and their Integrated Optical Density (IOD) was acquired by means of ImageJ 1.44p image analysis software. For the semi-quantification analysis, IOD of each sample was firstly normalized to the IOD of the loading control (ACTB), and then to the IOD of the positive control (MCF7 proteins for p38 β and p-p38, HepG2 proteins for CYP2B6, MAFF and UGT1A1).

### 5.10. Interleukin 6 Detection by ELISA

IL6 was quantified using the competitive Bovine IL6 Elisa kit, following manufacturer’s instructions. Each experimental condition (i.e., CTRL, 0.9 µM AFB1, 1.8 µM AFB1 and 3.6 µM AFB1) was analyzed in duplicate. The optical density (O.D.) was measured spectrophotometrically at 450 nm in a Multiskan Go multiwell plate reader (Thermo Fisher Scientific, Waltham, MA, USA).

### 5.11. CYP2B6 and NQO1 Enzymatic Activity

The catalytic activity of two enzymes significantly modulated at the mRNA level by AFB1, i.e., NQO1 and CYP2B6, was evaluated. For each experimental condition (i.e., CTRL, 0.9 µM AFB1, 1.8 µM AFB1 and 3.6 µM AFB1), six independent biological replicates were available and each of them was tested in duplicate (NQO1) or in sextuplicate (CYP2B6). NQO1 catalytic activity was measured as previously reported [117,119], while CYP2B6 enzymatic activity was measured using the P450-Glo™ CYP2B6 luminescent assay kit and VICTOR™X4 Multilabel Plate Reader (Perkin Elmer, Waltham, MA, USA). In both cases, the instructions provided by the manufacturer were followed. The luminescence was expressed in relative luminescence units (RLU) as the luminescence units normalized to the number of metabolically active cells obtained with CellTiter-Glo^TM^ cell viability assay kit (Section 5.3).

### 5.12. Statistical Analysis

Statistical analysis was performed using GraphPad Prism software (version 8.0.2, San Diego, CA, USA). One-way ANOVA followed by Bonferroni’s multiple comparisons test was used for the analysis of gene expression, enzymatic activity, ELISA and immunoblotting output data. For the assessment of apoptotic and necrotic cell rate, one-way ANOVA with Dunnett’s multiple comparisons test was implemented. A value of *p* < 0.05 was considered significant.

## Figures and Tables

**Table 1:** Concentration (µM) of AFB1, AFM1 and AFL measured in the cell medium after 48 h of incubation with increasing AFB1 concentrations (i.e., 0.9 µM, 1.8 µM and 3.6 µM). Data are expressed as the mean concentration ± standard deviation (SD) of four independent cell culture experiments.

	AFB1 (μM)	AFM1 (μM)	AFL (μM)
AFB1 0.9 μM	0.800 ± 0.037	0.002 ± 0.001	0.054 ± 0.004
AFB1 1.8 μM	1.170 ± 0.057	0.003 ± 0.001	0.074 ± 0.014
AFB1 3.6 μM	2.812 ± 0.183	0.006 ± 0.001	0.165 ± 0.020

## Data Availability

Raw Illumina Sequencing Data have been deposited in GenBank (SRA) under the BioProject ID PRJNA847423. Private access to the data is available at the link https://dataview.ncbi.nlm.nih.gov/object/PRJNA847423?reviewer=97vn2eecdlucsqkop4rsru09rl, accessed on 9 June 2022.

## Supplementary Materials

The following supporting information can be downloaded at: https://www.mdpi.com/article/10.3390/toxins14070504/s1, Table S1: Results of RNA sequencing, filtering and mapping; Table S2: list of DEGs for each treatment; Table S3: Over-representation analysis (KEGG pathways); Table S4: Gene Set Enrichment Analysis (KEGG pathways); Table S5: Log fold change (logFC) for each gene of interest; Figure S1: MDS plot; Figure S2: Number of DEGs between each AFB1 treatment (i.e., 0.9 µM, 1.8 µM, 3.6 µM) vs. control; Figure S3: KEGG enrichment analysis of the top PPI module; Figure S4: Effect of increasing doses of AFB1 (i.e., 0.9 µM, 1.8 µM and 3.6 µM) on FOS (A), FOSL1 (B) and FOSB (C) mRNA expression; Figure S5: Effect of increasing doses of AFB1 (i.e., 0.9 µM, 1.8 µM and 3.6 µM) on JUNB mRNA expression; Figure S6: Effect of increasing doses of AFB1 (i.e., 0.9 µM, 1.8 µM and 3.6 µM) on NFKB2 (A) and RELB (B) mRNA expression; Figure S7: Effect of increasing doses of AFB1 (i.e., 0.9 µM, 1.8 µM and 3.6 µM) on UGT1A1 mRNA and protein expression; Figure S8: Effect of increasing doses of AFB1 (i.e., 0.9 µM, 1.8 µM and 3.6 µM) on CYP2B6 mRNA level, catalytic activity and protein expression.
