# Supplementary material for: Deepening the Whole Transcriptomics of Bovine Liver Cells Exposed to AFB1: A Spotlight on Toll-like Receptor 2
Source: Toxins (Basel). 2022 Jul 20;14(7):504. doi: 10.3390/toxins14070504 (PMC9323327; doi:10.3390/toxins14070504)
Supplement: Supplementary file 1 [file toxins-14-00504-s001.zip › toxins-1806326-supplementary ms.pdf]

Supplementary Materials: Deepening the Whole Transcriptomics of Bovine Liver Cells Exposed to AFB1: A Spotlight on Toll-like Receptor 2

Silvia Iori, Marianna Pauletto, Irene Bassan, Federico Bonsembiante, Maria Elena Gelain, Anisa Bardhi, Andrea Barbarossa, Anna Zaghini, Mauro Dacasto and Mery Giantin

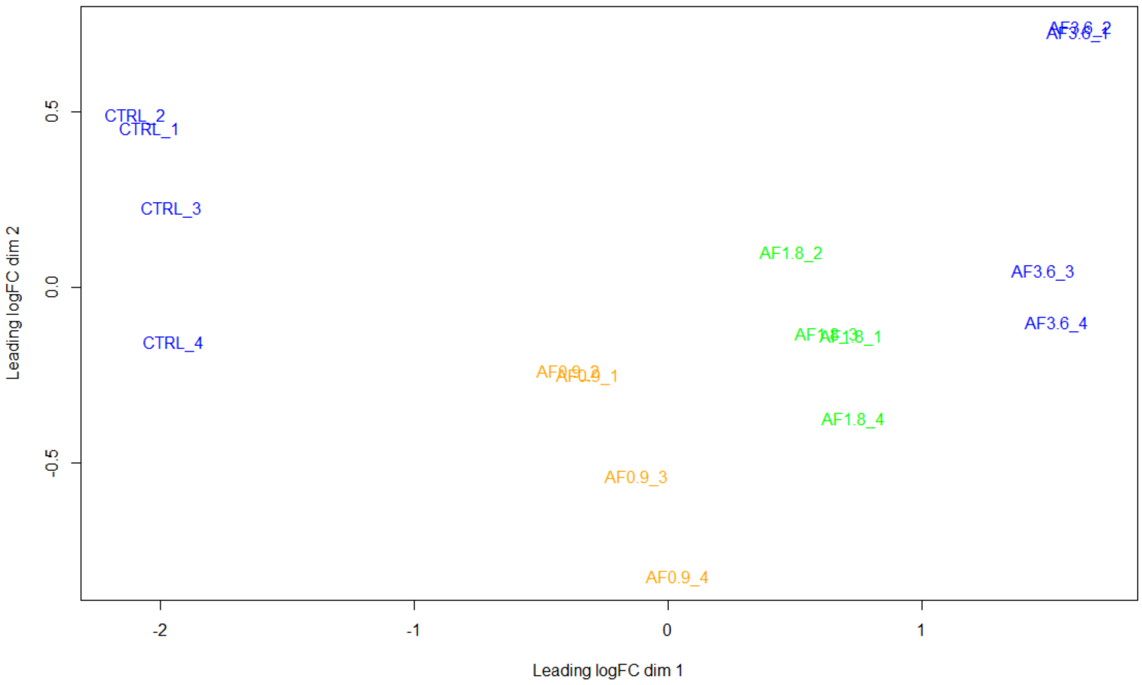

Figure S1. MDS plot. The plot reports distances between the expression profile of each library.

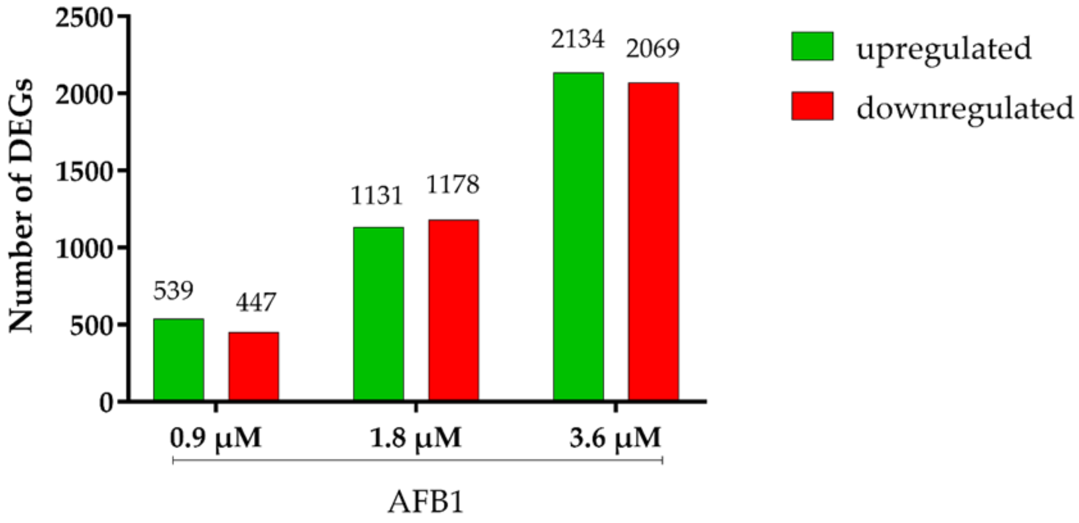

Figure S2. Number of Differentially expressed genes (DEGs) between each AFB1 treatment (i.e., 0.9 µM, 1.8 µM, 3.6 µM) vs control. Green colour represents upregulated genes, while red colour represents downregulated genes.

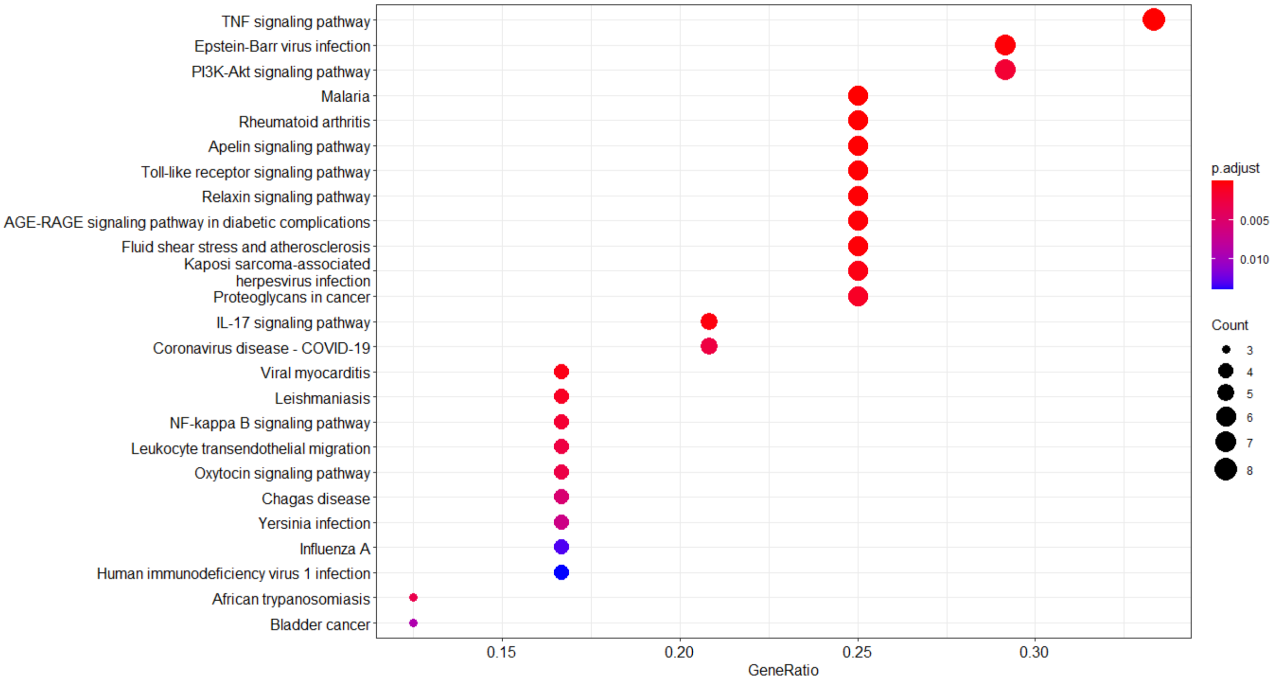

Figure S3. KEGG enrichment analysis of the top PPI module.

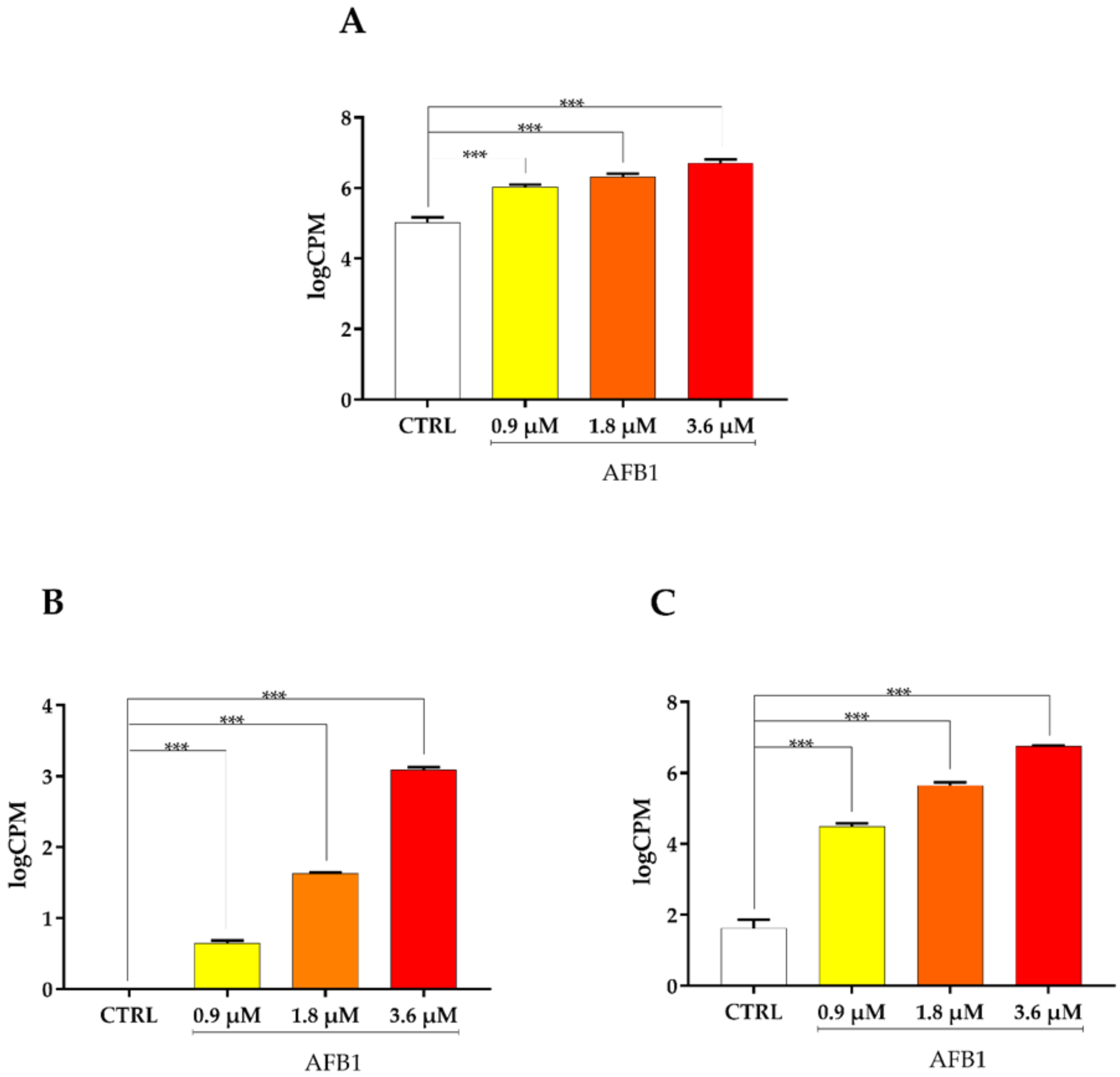

Figure S4. Effect of increasing doses of AFB1 (i.e., 0.9 µM, 1.8 µM, 3.6 µM) on FOS (A), FOSL1 (B) and FOSB (C) mRNA expression. Data are reported as the mean ± SEM of logCPM relative to four biological replicates. Statistical analysis: one-way ANOVA followed by Bonferroni's multiple comparisons test; ***: *P* < 0.001, treated cells vs control cells. LogCPM <0 was reported as 0.

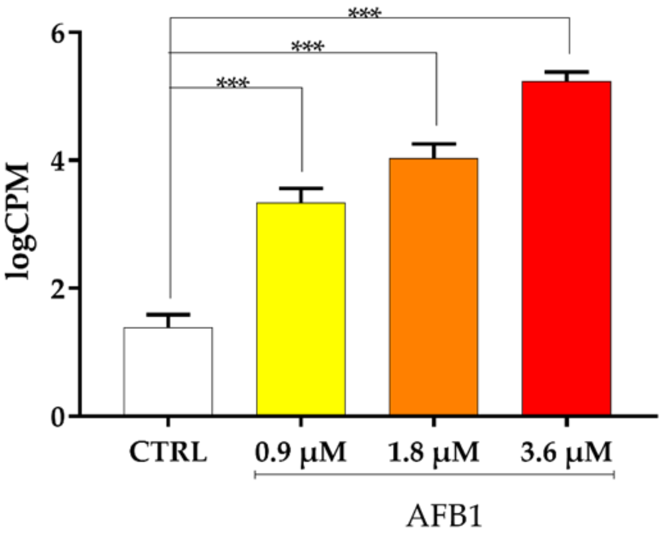

Figure S5. Effect of increasing doses of AFB1 (i.e., 0.9 µM, 1.8 µM, 3.6 µM) on JUNB mRNA expression. Data are reported as the mean ± SEM of logCPM relative to four biological replicates. Statistical analysis: one-way ANOVA followed by Bonferroni's multiple comparisons test; ***: *P* < 0.001, treated cells vs control cells.

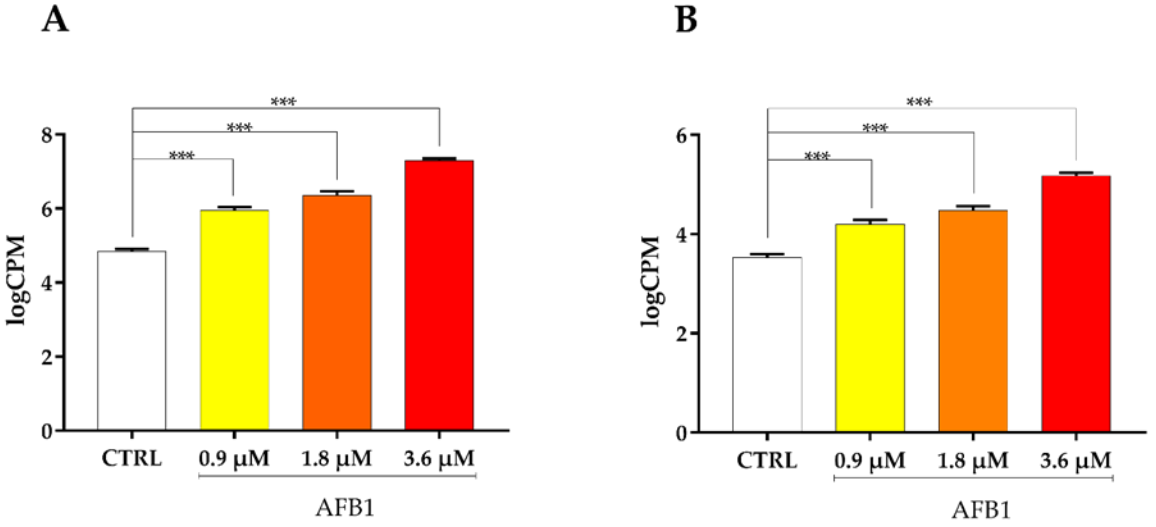

Figure S6. Effect of increasing doses of AFB1 (i.e., 0.9 µM, 1.8 µM, 3.6 µM) on NFKB2 (A) and RELB (B) mRNA expression. Data are reported as the mean ± SEM of logCPM relative to four biological replicates. Statistical analysis: one-way ANOVA followed by Bonferroni's multiple comparisons test; ***: *P* < 0.001, treated cells vs control cells.

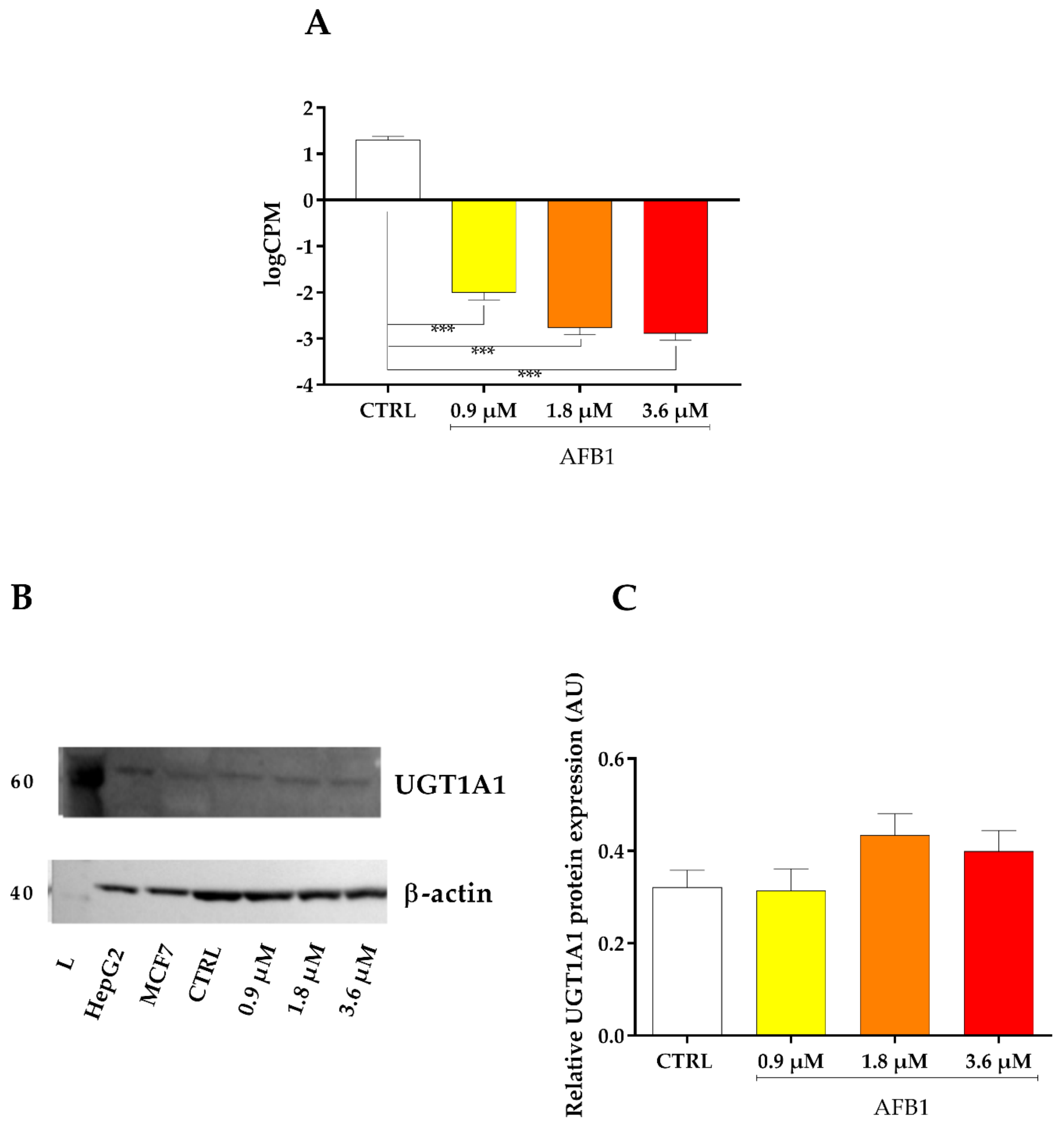

Figure S7. Effect of increasing doses of AFB1 (i.e., 0.9 µM, 1.8 µM, 3.6 µM) on UGT1A1 mRNA and protein expression. (A) Gene expression data are reported as the mean ± SEM of logCPM relative to four biological replicates. (B) UGT1A1 immunoblotting, using β-actin as loading control. The image is representative of six biological replicates. HepG2 and MCF7 cell lines were used as positive controls. (C) Densitometric analysis of UGT1A1 immunoblottings; results are expressed in arbitrary units (AU) as the mean ± SEM of six biological replicates. Statistical analysis: one-way ANOVA followed by Bonferroni's multiple comparisons test; ***: $P < 0.001$, treated cells vs control cells.

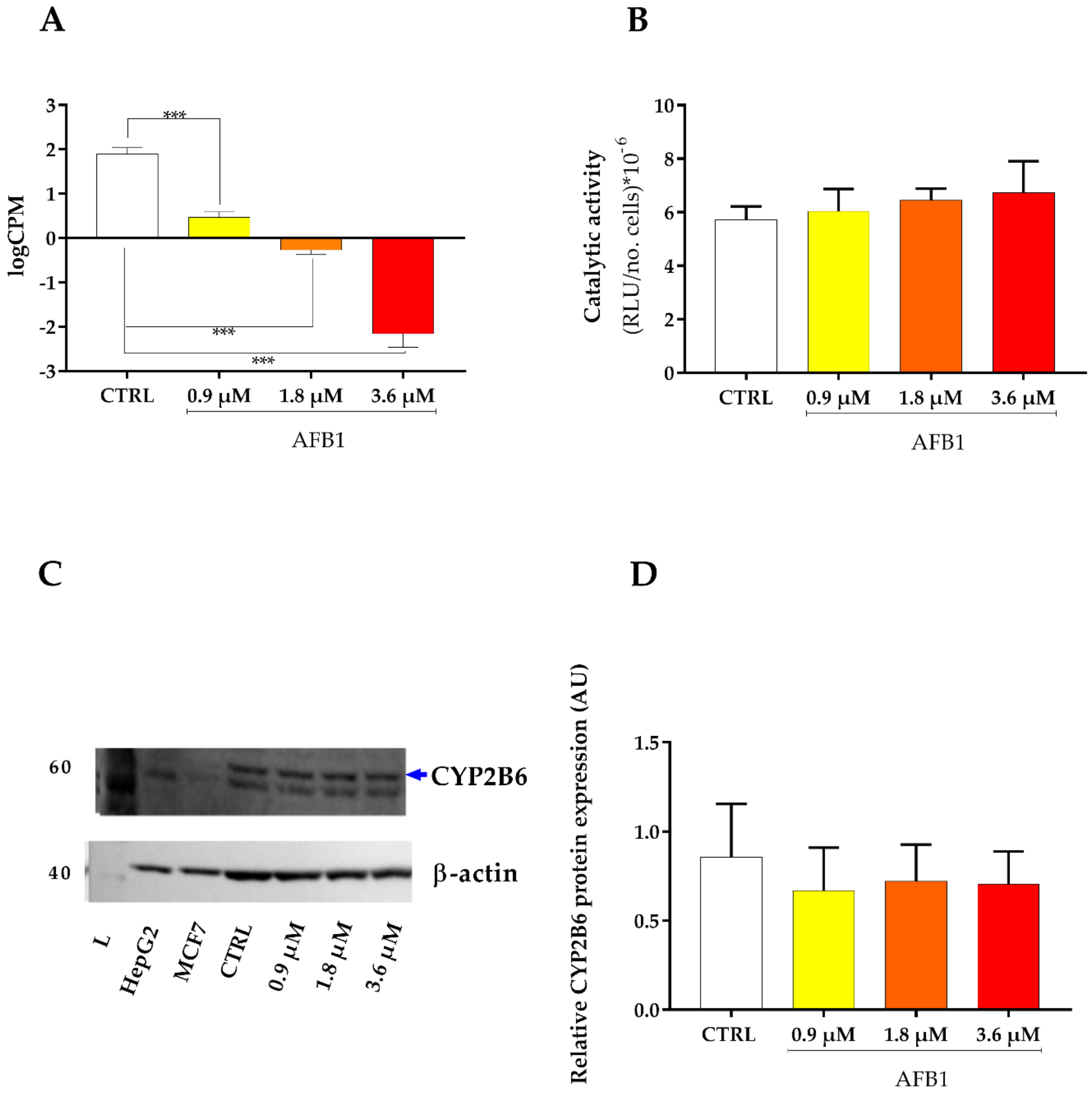

Figure S8. Effect of increasing doses of AFB1 (i.e., 0.9 µM, 1.8 µM, 3.6 µM) on CYP2B6 mRNA level, catalytic activity and protein expression. (A) Gene expression data are reported as the mean ± SEM of logCPM relative to four biological replicates. (B) Catalytic activity is expressed as relative luminescence units (RLU), such as luminescence units normalized to the total number of alive cells. (C) CYP2B6 immunoblotting, using β-actin as loading control. The image is representative of six biological replicates. HepG2 and MCF7 cell lines were used as positive controls. (C) Densitometric analysis of CYP2B6 immunoblotting; data are expressed in arbitrary units (AU) as the mean ± SEM of six biological replicates. Statistical analysis: one-way ANOVA followed by Bonferroni's multiple comparisons test; ***: $P < 0.001$, treated cells vs control cells.
